# Converting focused ultrasound–based boiling histotripsy into a systemic cancer vaccine using antigen-capturing microparticles

**DOI:** 10.7150/thno.132091

**Published:** 2026-05-18

**Authors:** Akansha Singh, Sri Vidhya Chandrasekar, Faraz Chamani, Harshini Ashar, Steve Hartson, Steve Fiering, Ashish Ranjan

**Affiliations:** 1 Department of Radiation Oncology, UT Southwestern Medical Center, Dallas, TX, 75390 (Current affiliation), USA.; 2 Department of Physiological Sciences, College of Veterinary Medicine, Oklahoma State University, Stillwater, OK 74078, USA.; 3 Department of Biochemistry, Oklahoma State University, Stillwater, OK 74078, USA.; 4 Geisel School of Medicine, Dartmouth, Hanover, NH03755, USA.

**Keywords:** boiling histotripsy, microparticles, CD40 agonistic antibody, abscopal effect, immune checkpoint inhibitors, cold tumors

## Abstract

**Background:**

Tumors with an immunosuppressive tumor microenvironment (TME) limit effective anti-tumor immunity. Focused ultrasound–based boiling histotripsy (HT) is a noninvasive technique that mechanically ablates tumors while generating acellular lysates with preserved tumor antigens in situ. However, HT alone elicits insufficient anti-tumor immune activation to induce regression of both local and metastatic tumors, particularly in immunoresistant settings. To address this limitation, the objective of this study was to engineer biodegradable polymeric microparticles (MPs) loaded with an agonistic CD40 antibody (CMP) to capture HT-released tumor antigens, enabling sustained uptake and presentation by antigen-presenting cells (APCs) while activating dendritic cells (DCs) to enhance APC priming.

**Methods:**

Microparticles were synthesized via a water-in-oil-in-water double emulsion method using PLGA/PCL polymers and an agonistic CD40 antibody. Boiling HT was delivered using the Alpinion HIFU system (VIFU 2000) with a 1.5 MHz transducer, millisecond pulses at 5 Hz, 1% duty cycle, and 600 W power to generate antigen-rich lysates in murine tumors at low (<10%) and high (>50%) ablation. Multiple murine tumor models (melanoma, head and neck, colon) were used to assess local efficacy, immunomodulation, and abscopal responses following HT+CMP therapy. Additionally, in vitro HT lysate adsorbed onto CMPs was administered subcutaneously as a vaccine to promote systemic immunity.

**Results:**

Across multiple murine tumor models, local HT+CMP therapy elicited robust local and abscopal immune responses at both low and high ablation volumes. These were associated with increased functional immune cell infiltration and reversal of resistance to immune checkpoint inhibitors (ICIs). Similar effects were observed using in vitro HT-generated lysate adsorbed onto CMPs and administered subcutaneously as a vaccine to promote systemic immunity.

**Conclusions:**

By coupling sustained antigen availability with enhanced APC activation, HT-CMP therapy represents a promising strategy to achieve local and abscopal immune responses and overcome ICI resistance, particularly in immunologically "cold" tumors.

## Introduction

Immunologically "cold" tumors are characterized by a paucity of tumor-infiltrating lymphocytes (TILs) and exhibit low intrinsic immunogenicity, rendering them less responsive to treatments that depend on T cell-mediated antitumor immunity, such as immune checkpoint inhibitors (ICIs) 1-3. To overcome these barriers, energy-depositing modalities such as photothermal therapy (PTT), alternating magnetic fields (AMF), focused ultrasound (FUS), and ionizing radiation are emerging as promising approaches. These modalities aim to remodel the immunosuppressive tumor microenvironment (TME) by promoting immunogenic tumor antigen release and the expression of damage-associated molecular patterns (DAMPs), including calreticulin membrane exposure and ATP/HMGB1 release. These signals enhance antigen uptake and presentation by antigen-presenting cells (APCs) and promote T cell priming, while also inducing inflammatory cytokine and chemokine secretion and transient vascular and stromal changes that facilitate immune cell trafficking and reduce myeloid- and stroma-mediated immunosuppression 4-8. Among these, non-invasive FUS offers precise energy deposition under image guidance, enabling high spatial accuracy and adaptability across various tumor sites 9-12. FUS precision and versatility have led to FDA approval for multiple tumor indications, including uterine fibroids, prostate cancer, and pain palliation for bone metastases 13. These approvals highlight the clinical potential of FUS as a safe and effective modality for tumor-targeted therapies, further supporting its combination with immunotherapeutic strategies to overcome the challenges associated with cold tumors.

Of particular interest is histotripsy (HT), a non-thermal ultrasound technique that uses mechanical shockwaves at low duty cycles to ablate tumors by generating cell lysates 14-17. Clinical applications of cavitation-based HT, which relies on bubble clouds generated by short, microsecond high-pressure pulses, have shown promising outcomes, including recent hepatic trials 18. In contrast, boiling HT, which employs higher ultrasound transducer frequencies (~1-3 MHz) and millisecond-long pulses to nucleate vapor bubbles and generate antigen-rich lysates within the TME 19-23, has been shown by us and others to enhance anti-tumor immune responses in preclinical models of melanoma, breast cancer, and neuroblastoma 24-26. However, the antigenic effects of HT alone can be transient, and systemic anti-tumor immunity is often modest, as released tumor antigens within the TME are susceptible to rapid proteolytic degradation or clearance before effectively engaging APCs 27. Moreover, the immunosuppressive TME characterized by high levels of regulatory T cells (Tregs), myeloid-derived suppressor cells (MDSCs), and inhibitory cytokines further impairs antigen processing and presentation. Collectively, these barriers limit the immune system’s ability to mount a durable and effective anti-tumor response, thereby constraining the therapeutic potential of HT and related modalities.

We hypothesized that intratumorally administered microparticle (MP)-based delivery systems, engineered to capture and preserve tumor lysates released during HT within the TME, could prevent their degradation and provide sustained antigen availability, thereby extending the presentation window to APCs. However, sustained antigen release alone is insufficient to overcome the profound immune suppression characteristic of immunologically “cold” tumors. Effective immunotherapy also requires robust activation of APCs, including dendritic cells (DCs), macrophages, and B cells 28, 29. Among these, DCs are uniquely capable of priming naïve T cells and initiating durable antitumor responses. Yet in cold tumors, APC activation is frequently suppressed, creating a hostile microenvironment that limits immune engagement 30, 31. Activation of CD40 with agonistic αCD40 32 induces activated APC (dendritic cell & macrophage) phenotypes characterized by increased MHC-II and CD80/86 expression 33, enabling effective antigen presentation of tumor antigens and thereby enhancing T cell-mediated anti-tumor immunity 34. These unique properties have led to clinical trials of αCD40 for anti-tumor immune activation 35. Although promising, systemic administration of αCD40 agonists in patients has been associated with dose-limiting toxicities, including cytokine release syndrome, hepatotoxicity, and hematologic abnormalities such as thrombocytopenia, which constrain dosing and limit therapeutic efficacy. These toxicities largely arise from broad activation of CD40 expressed on circulating and tissue-resident immune cells, resulting in systemic immune activation rather than tumor-restricted stimulation. Even with Fragment crystallizable (Fc) engineering strategies designed to optimize FcγReceptor–mediated crosslinking and enhance agonistic activity, many CD40 antibodies retain a narrow therapeutic window and require careful dose escalation or local delivery strategies to mitigate off-target immune effects 36-39. Also, as a single agent, αCD40 largely achieves modest clinical benefits, and its efficacy depends upon the presence of an immunogenic environment and optimal immune priming – features not suited to treating cold tumors 40. Fc-engineered CD40 agonists have been developed to improve FcγReceptor-dependent crosslinking and reduce the systemic toxicities associated with CD40 therapy; however, despite encouraging preclinical results, these agents have yet to achieve widespread clinical use 41 and local delivery strategies are often needed to mitigate systemic adverse events 39, 42.

To address these barriers, we developed a combinatorial strategy that integrates boiling HT–induced antigen release with αCD40-encapsulating microparticles (CMPs). Synthetic polymer-based particles can adsorb proteins through formation of a non-covalent protein corona, enabling efficient capture of HT-released tumor antigens 43. Concurrently, CMPs capture tumor antigens following the HT lysis within the TME and provide sustained local release of both antigen and αCD40 to APCs, thereby promoting antigen uptake and MHC-I-mediated presentation. By coupling the spatial precision of FUS-based HT with sustained, particle-mediated immunomodulation, this approach is designed to overcome key immunological constraints across tumors with varying degrees of immunogenicity.

## Material and Methods

### Materials

50:50 Poly(DL-lactide-co-glycolide), ester terminated (PLGA) 0.15-0.25 dL/g in HF1P (#B6017-1G, Lactel) and Poly (ε-caprolactone), ester terminated (PCL) 0.65-0.85 dL/g in CHCL3 (#B6003-1P, Lactel) were purchased from DURECT Corporation (AL, USA). Poly(vinyl alcohol) 30,000-70,000 MW (PVA; #P8136), Dichloromethane (DCM; #270997), Deoxyribonuclease I from bovine pancreas (DNase I; #D4527), Liberase™ TM Research Grade (#5401119001), Dulbecco’s Phosphate Buffered Saline (PBS; #D8537), DMEM-High Glucose culture media (#D6429) were procured from Millipore-Sigma (MO, USA). Cetyltrimethylammonium bromide (CTAB; #0833) was purchased from Avantor-VWR (PA, USA). Murine GM-CSF (#315-03) was purchased from PeproTech (NJ, USA). InVivoMAb anti-mouse CD40 agonist antibody (#BE0016-2), InVivoMAb anti-mouse CTLA-4 (CD152) antibody (#BE0032), InVivoMAb anti-mouse PD-L1 (B7-H1) antibody (#BE0101) were acquired from BioXCell (NH, USA). Acetonitrile (#300000ACS) was purchased from Pharco-AAPER. Cell culture grade Water (#118-162-101) was procured from Quality Biologicals (MD, USA). Sodium Dodecyl Sulfate (SDS; #BP166, Fisher Bioreagents), D-Sucrose (#BP220-1, Fisher Bioreagents), Rat IgG2a isotype control (#02-9688), Pierce™ Rapid Gold BCA Protein Assay Kit (#A53225), Fluoraldehyde™ o-Phthaldialdehyde (OPA) Reagent Solution (#26025), Pierce™ BCA Protein Assay Kit (#23227), RPMI media (11875093), Fetal bovine serum, FBS (10082147), Penicillin-Streptomycin, PenStrep (15140122), Versene Solution (#15040066), 2-Mercaptoethanol (#21985023), Albumin from Bovine Serum (BSA), FITC conjugate (#A23015), eBioscience™ 10X RBC Lysis Buffer (#00-4300-54), eBioscience™ Brefeldin A solution 1000X (#4506-51), Trypan blue 0.4% solution (#15250061), LIVE/DEAD fixable dead cell stains (#L34965, #L34964 & #L34971) were purchased from Gibco/Invitrogen/Thermo Fisher Scientific (MA, USA). BD Pharmingen™ Transcription Factor Buffer Set (#562574) was procured from BD Biosciences (CA, USA). 4–20% Mini-PROTEAN® TGX™ Precast Protein Gels (#4561094) were purchased from BioRad (CA, USA). TRP-2 (AS-61058) and AH1 (AS-64798) peptide were procured from Anaspec (CA, USA).

Anti-mouse flow cytometry antibodies: APC-Cy7 anti-CD45 (#557659), Pe-Cy7 anti-CD45 (#552848), BV785 anti-CD4 (#565634), BB515 anti-MHC-II (#565254), BV480 anti-MHC-II (#566086), BV421 anti-CD40 (#562846) & BB700 anti-MHC-I (#749699) were purchased from BD Biosciences, San Jose, CA, USA. PerCP anti-CD3 (#100326), PE-Cy5 anti-CD3 (#100273), BV785 anti-CD4 (#100453), PE-Cy7 anti-CD8 (#100722), BV421 anti-CD8 (#100738), BV785 anti-CD279 (PD1; #135225), BV650 anti-CD44 (#103049), PE-Cy5 anti-IL2 (#503824), PE anti-Ly6C (#128008), AF647 anti-Ly6G (#127610), PE-Cy7 anti-CD172a (SIRPα; #144008), APC-Cy7 anti-CD11c (#117324), FITC anti-MHC-II (#107605), PE anti-CD86 (#105008), BV785 anti-CD86 (#105043), AF-488 anti-CD40 (#102910), BV650 anti-CD11b (#101259), PE-Cy5 anti-CD206 (#141740), AF647 anti-IFNγ (#505814) were procured from BioLegend, San Diego, CA, USA. PE-Texas Red anti-CD11c (#MCD11C17), PE anti-GranzymeB (#12-8898-82), AF488 anti-FOXP3 (#53-5773-82), PE-Texas Red anti-CD62L (#RM4317) were purchased from Invitrogen, Waltham, MA, USA. Mouse melanoma cell line, B16F10 (RRID:CVCL_0159) & B16F10-Ova (#CRL-6475) was a gift from Dr. Turk at Dartmouth College. Colorectal carcinoma cell line, CT26 (#CRL-2638; RRID:CVCL_7256) were purchased from ATCC (VA, USA). Mouse OSCC (MOC2; RRID:CVCL_ZD33) cell line (#EWL002-FP) was purchased from Kerafast, Boston, MA, USA. Mouse cell lines were cultured in standard DMEM media supplemented with 10% fetal bovine serum (FBS) and 1% penicillin-streptomycin. Cell lines were routinely tested for mycoplasma contamination and authenticated using STR profiling. 7-8 weeks old Male or Female C57BL/6 (RRID:IMSR_JAX:000664) and BALB/c mice (RRID:MGI:2683685) were purchased from Charles River Laboratories. All animal experiments were conducted under protocol (#21-85) approved by the IACUC at Oklahoma State University, adhering to ethical guidelines for the care and use of laboratory animals.

### Method

#### Microparticle preparation and characterization

Microparticles were synthesized using a water-in-oil-in-water double emulsion solvent evaporation technique. The organic phase consisted of PLGA and PCL dissolved in DCM at a concentration of 50 mg/mL. Mixtures of PLGA and PCL were prepared at specific v/v ratios (100:0, 75:25, 50:50, 25:75, 0:100). 100 μL of internal aqueous phase (IAP) containing IgG isotype or αCD40 antibody, along with other excipient mentioned in Table 1, was added to the organic phase (OP) and sonicated on ice for 2 min using a Branson Sonifier 450. This primary emulsion was then transferred into 40 mL of external aqueous phase (EAP) containing 0.1% CTAB and homogenized at 10,000 rpm for 10 min at room temperature using a Benchmark’s D1000 homogenizer. The resulting particles were stirred overnight at room temperature to allow solvent evaporation, washed three times with MilliQ water by centrifugation at 10,000 rpm, and lyophilized using a Labconco FreeZone lyophilizer for long-term storage. Microparticle size and morphology were assessed using dynamic light scattering (DLS) on a Malvern Mastersizer 2000 and scanning electron microscopy (SEM) on a JEOL JSM-IT500 system.

An unconjugated IgG (150 kDa) isotype was initially used to assess antibody loading in PLGA-PCL MPs for optimizing encapsulation across varying ratios. Antibody loading of various microparticle formulations were estimated by dissolving 25 mg microparticles entrapping IgG isotype control in acetonitrile followed by 1 h incubation at constant shaking. Suspension was centrifuged at 13,000 RPM, 10 min, 4 ºC (Microfuge 5418, Eppendorf, CT, USA) and pellet was resuspended again in acetonitrile to repeat the process three times. Final pellet was resuspended in PBS with 1% SDS. Protein estimation was done using micro-BCA protein assay kit following manufacturer protocol. Absorbance of appropriate blank microparticles were subtracted from IgG control microparticles before calculating loading.

Practical load of microparticles was calculated using following formula:

Practical load (μg/mg) = Protein amount estimated (μg)/Amount of particles used (mg)

Theoretical load was calculated using amount of IgG taken in IAP per mg of polymer used in OP:

Theoretical load (μg/mg) = IgG added to IAP (μg)/Polymer amount in OP (mg)

Because loading was quantified from a fixed aliquot of each batch (25 mg) to account for batch-to-batch variability and to guide dosing in downstream experiments, we report a batch-normalized Loading realization (%) metric:

Loading realization (%) = (Practical load/Theoretical load) * 100

In vitro release of entrapped IgG from various microparticle formulations were estimated using Fluoraldehyde o-Phthaldialdehyde (OPA) assay. In brief, 200 mg of IgG isotype control microparticles as well as blank microparticles were resuspended in 1mL of PBS in microfuge tubes and incubated at 37 ºC at 200 RPM in orbital shaker. At different times (1 h, Day-1, 3, 5, 15 & 30), 20 μl supernatant was removed from tubes. In 96-well plate, 20 μL of collected supernatant, blank and standards were mixed with 200 μL of OPA reagent in dark and fluorescence (excitation- 380 nm, emission- 450 nm) was measured within 5 min of mixing.

To analyze structural stability of released αCD40 antibody from PLGA:PCL 50-50 microparticles, protein intrinsic fluorescence was analyzed. Briefly, 50 mg of CMP were resuspended in 1 mL PBS and incubated at 37 ºC at 200 RPM in orbital shaker for 24 & 48 h. Supernatant was collected by centrifuging suspension at 12,000 RPM for 10 min (Microfuge 5418, Eppendorf, CT, USA) and fluorescence was measured using Cary Eclipse fluorescence spectrophotometer (Agilent, CA, USA). Collected supernatants and 20 μg/ml αCD40 antibody solution were excited at 280 nm and emission spectra were collected from 300-420 nm with excitation and emission slit width set at 5 nm.

#### Bone marrow-derived dendritic cell (BMDC) stimulation assay

Bone marrow cells were harvested from the femurs and tibias of 10-week-old female C57BL/6 mice under sterile conditions. Cells were cultured in RPMI-1640 media supplemented with 10% FBS, 1% penicillin-streptomycin, 50 μM β-mercaptoethanol, and 20 ng/mL GM-CSF to promote differentiation into dendritic cells. On day 5, non-adherent and loosely adherent cells were collected and seeded into 24-well plates at a density of 0.1 × 10^6^ cells per well. Cells were treated with tumor lysates generated by sonicating 10,000 B16F10 cells in PBS on ice for 2 min using Branson Sonifier 450. Cell suspension was centrifuged at 12,000 RPM at 4 ºC to remove partially lysed cell and membrane fragments for protein fraction. Treatments included free αCD40 antibody or αCD40 encapsulated in CMPs, both at a final concentration of 10 μg/mL. After 48 h, cells were harvested, washed, and trypan blue exclusion assay was performed. >90% of live BMDCs were stained with antibodies targeting activation markers CD40, CD86, MHC-I and MHC-II. Flow cytometry analysis was performed to assess dendritic cell activation, using a BD LSR II system with compensation controls to establish gating thresholds.

#### Protein adsorption on MPs

To evaluate protein adsorption capacity, blank microparticles were incubated with FITC-conjugated bovine serum albumin (BSA) at a concentration of 5 μg/mL for 1 h at room temperature under constant agitation. Unbound proteins were removed by washing the particles three times with PBS. Adsorbed proteins were visualized using an Olympus IX81 inverted Epi-fluorescence microscope. Quantification of adsorbed protein was performed by incubating blank microparticles in FBS (concentration- 1.5, 3.125, 6.25, 12.5, 25, 50 & 100%) for various time points, 0.5, 1, 2, 4 and 12 h at RT. Particles were collected by centrifugation at 12,000 RPM, 15 min, 25 ºC and washed thrice with PBS. Surface adsorbed proteins were extracted with repeated acetonitrile washes as mentioned in earlier and quantified using Pierce BCA protein assay kit.

Release profile of surface adsorbed protein was evaluated by resuspending 20mg of lyophilized blank microparticles in 50% FBS (diluted in PBS) and incubating for 2 h at room temperature. Following three PBS washes, protein adsorbed particles were incubated 37 ºC at 200 RPM in orbital shaker for various time points, 1, 3, 6 and 9 days. Supernatant was collected from suspension by centrifugation at 12,000 RPM, 10 min, 4 ºC and released protein was estimated using Pierce BCA protein assay kit. Released proteins were also visualized by running 20 μl of supernatant on SDS-PAGE (Mini-PROTEAN Tetra, Biorad, CA, USA).

To characterize tumor protein adsorption on particles mediated by FUS-HT in vitro, 15 X 10^6^ B16F10 tumor cells and CT26 tumor cells in serum free media in thin-walled PCR tubes underwent HT treatment. Histotripsy was performed using the Alpinion FUS transducer (1.5 MHz) with a 45 mm radius of curvature and 64 mm aperture diameter (central opening: 40 mm). The focal zone dimensions (~10 × 1 mm) were defined based on the manufacturer-reported focal region corresponding to the -6 dB pressure field (i.e., full width at half maximum, FWHM) along the axial and lateral axes. The following parameters were used: electrical input power, 600 W; pulse repetition frequency (PRF), 5 Hz; duty cycle, 1%; pulse duration, 2 ms; and treatment time per focal point (dwell time), 20 s (100 pulses). Based on system specifications and previously reported measurements for similar 1.5 MHz histotripsy transducers, we estimate that these settings generated peak negative pressures of ~15–25 MPa and peak positive pressures of ~40–80 MPa, consistent with boiling histotripsy conditions. Protein released from lysed cells (Treatment supernatant) was separated from live cells that survived HT treatment as well as insoluble debris by centrifuging at 1200 RPM, 5 min, 4 ºC. Treatment supernatant was named as BT: B16F10 cells & CT: CT26 cells. Surviving cells releasing stress associated antigens overtime were cultured in serum free DMEM media for another 48 h and the culture supernatant was collected separately. Culture supernatant was named BC: B16F10 cells & CC: CT26 cells. Collected supernatants were incubated with 20 mg of blank particles separately for 2 h at RT under constant stirring, 80 RPM. Following three PBS washes particle pellet was resuspended in 500 μl PBS and then added to 2.5 mL of acetonitrile and mixed at 45 ºC for 3 h under constant shaking, 250 RPM, to dissolve polymer. To separate proteins from polymer in the mix, acetonitrile was evaporated under vacuum and remaining aqueous solution with solubilized protein and aggregated polymers was centrifuged at 1500 RPM, 10 min, 25 ºC. Pellet were discarded and supernatant with retrieved proteins were used for LC-MS-MS analysis as previously published 44. Sample processing, data acquisition, data processing and protein quantification was done at the Proteomics Core Facility at Oklahoma State University. Briefly, proteins from CT26- and B16-derived microparticles were digested, reconstituted in 0.1% formic acid, clarified, and analyzed by triplicate nanoLC–MS/MS injections (Orbitrap Fusion, C18 75 µm × 50 cm, 2 h gradient). Data-dependent acquisition was performed with HCD fragmentation, and peptide/protein identification used MaxQuant against the Mus musculus UniProt database. Differential analysis was conducted as previously described. Further, B16F10 cell treatment supernatant was adsorbed and processed as above and visualized using SDS-PAGE.

#### *In vivo* tumor models and histotripsy treatments

All mice were housed and treated following protocol (#21-85) approved by IACUC, Oklahoma State University, Oklahoma. For tumor inoculations, all mouse cancer cell lines were cultured in DMEM containing 10% v/v fetal bovine serum (FBS) and 1% v/v streptomycin/penicillin. Cells were harvested at 70-80% confluency, rinsed and diluted in sterile cold PBS before injecting in mice.

Dose-comparison study designs: To assess whether CMP reduces dosing frequency, we compared three administrations of free αCD40 with a single administration of CMP-delivered αCD40 (αCD40^3^:CMP 1), where a 3:1 ratio denotes three administrations of free αCD40 for every one CMP administration.

**3:1 dosing frequency study in B16F10 tumors**: 7-8 weeks old C57BL/6 mice were inoculated subcutaneously in the right flank with 0.5 × 10^6^ B16F10 melanoma cells in 100 µL of PBS using a 27-gauge needle (BD, Franklin Lakes, NJ, USA). Mice tumor volume was measured daily by serial caliper measurements (General Tools Fraction™, New York, NY, USA) using the formula (length × width^2^)/2, where length was the largest dimension and width was the smallest dimension perpendicular to the length. When the average volume of B16F10 tumors reached ~100 mm^3^, mice were divided into 4 groups (n = 6 per group, males & females randomized), 1) Vehicle control, 2) αCD40, 3) Isotype antibody entrapping MP (BMP), & 4) CMP. Free αCD40 (30 μg) diluted in PBS was administered intratumorally (I.T.) three times at three-day intervals using a 27-gauge needle. CMP-treated mice received a single I.T. injection of CMP containing 30 μg αCD40 using a 25-gauge needle. BMP-treated mice received an I.T. injection of isotype control antibody entrapping BMP equivalent in weight to CMP (Schematic in result figure). Tumor volumes were monitored daily, and mice were sacrificed when tumors exceeded 2000 mm³.

**1:1 matched dose comparison in MOC2 tumors**: Head and neck squamous cell carcinomas (MOC2) were established by subcutaneous inoculation of 0.15 × 10^6^ MOC2 cells. When tumors reached ~100 mm^3^, mice were randomized as above and treated with either free αCD40 (30 μg, diluted in PBS) or CMP containing 30 μg αCD40, administered intratumorally once per week for a total of two treatments (Schematic in result figure). The weekly dosing schedule was based on the in vitro release profile of CMP-encapsulated αCD40. All mice were sacrificed 4 days after the final treatment (day 27 post-inoculation), tumor draining lymph nodes (TDLNs) and tumor samples were collected in DMEM supplemented with FBS for flow cytometry analysis.

**Evaluate abscopal effects and early immune activation signatures:** Bilateral tumor models of B16F10 and CT26 colon cancer were established by inoculating secondary untreated contralateral tumors in the left flank 3 days later of primary tumor inoculation in right flank to evaluate abscopal effects and early immune activation signatures. 7-8 weeks old female C57BL/6 mice were inoculated subcutaneously with 0.5 × 10^6^ B16F10 cells and 1 × 10^6^ CT26 cells in 7-8 weeks old female BALB/c mice. 8 days post-inoculation when primary tumors were >80 mm^3^ volume, mice were randomized in 6 treatment groups (n = 6), 1) Control, 2) HT, 3) αCD40, 4) CMP, 5) αCD40+HT, 6) CMP+HT. αCD40 and CMP treatment was given as above, for αCD40+HT and CMP+HT, αCD40 and CMP were injected immediately after HT at the site of treatment. Boiling histotripsy was performed using an Alpinion HIFU system, wet-type VIFU 2000 with 1.5 MHz HIFU transducer, with parameters optimized for tumor homogenization (600 W electrical power, 5 Hz pulse repetition frequency, 1% duty cycle) as we previously published 45. Primary (treated) tumors were aligned at a fixed focal volume (1 × 1 × 10 mm) under ultrasound guidance, and HT was delivered for 20 s per focal point to cover either ~10% (low HT) or >50% (high HT) of tumor volume. HT was delivered at the tumor core-periphery interface. The treatment path was defined as a 1D raster of focal region along the X-axis (horizontally), with adjacent focal points placed with 0.5 mm overlap to ensure contiguous coverage. The focus was advanced mechanically between focal points using a motorized positioning system (2D stage), while treatment stepping was performed along X-axis to cover the planned sub-volume. HT treatment parameters are summarized in Supplementary Table S1. Bubble formation was confirmed by ultrasound imaging. Tumors were monitored daily, and all mice were euthanized on day 8 for immunological analysis. The day 8 endpoint was chosen to allow sufficient activation of both innate and adaptive immune responses. Treated and abscopal tumors along with TDLNs were collected in DMEM for immune cell analysis using flow cytometry. Blood was collected for serum cytokine measurements, and spleens were harvested in RPMI medium for ex vivo splenocyte stimulation assays to evaluate tumor-specific CD8+ T cell activation.

To compare reversal of ICI resistance across treatments in murine melanoma, bilateral subcutaneous B16F10 and B16F10-OVA tumors were established as described above. When average tumor volume reached 80 mm^3^, male and female 7-8 weeks old C57BL/6 mice were divided into 12 treatment groups (n = 5-6 for B16F10-OVA, n = 6 for B16F10), 1) Control, 2) ICI, 3) αCD40, 4) CMP, 5) αCD40+ICI, 6) CMP+ICI, 7) HT, 8) HT+ICI, 9) αCD40+HT, 10) CMP+HT, 11) αCD40+HT+ICI, 12) CMP+HT+ICI. B16F10-bearing mice received two HT treatments, as described above, followed by intratumoral injections of αCD40 or CMP on days 8 and 19 post-inoculation. In contrast, B16F10-OVA–bearing mice developed surface necrosis after the first HT treatment (day 8) in several groups; therefore, on day 19, these mice only received intratumoral αCD40 or CMP injections to avoid further ulceration. To establish ICI resistance in B16F10 tumor bearing mice, six doses of immune checkpoint inhibitors were administered intraperitoneally: 75 μg anti–CTLA-4 and 150 μg anti–PD-L1, starting the day after HT (days 9, 11, 13 and 20, 22, 24 post-inoculation). B16F10-OVA tumor bearing mice received six ICI treatments intraperitoneally every other day from day 9 to day 19 post-inoculation. Mice were monitored daily for tumor growth and signs of ulceration and euthanized if tumors exceeded 1500 mm^3^ volume or if severe ulceration was observed.

#### CMP vaccination studies

Vaccination studies were designed to evaluate systemic immune responses elicited by CMPs adsorbed with tumor-associated antigens generated by histotripsy. Because monotherapies such as free αCD40, BMPs, or CMPs encapsulating αCD40 showed limited efficacy in B16F10 and MOC2 models, they were not included as controls. Instead, the focus was on delivering HT-generated tumor proteins with CMPs in the presence of ICIs. Tumor lysates were generated from histotripsy-treated B16F10 cells in vitro and incubated with CMPs at a ratio of 1 mg of CMPs per 100 μg of tumor lysate protein for 2 h at room temperature. Female C57BL/6 mice were vaccinated subcutaneously in the right flank with 100 μL of antigen-loaded CMPs on days 0, 2, and 4. Control groups included free tumor lysates, ICI, and vehicle alone groups. On day 7, all mice were challenged with a subcutaneous injection of 0.5 × 10^6^ B16F10 cells in the left flank. 3 ICI treatments, including 75 µg anti-mouse CTLA4 and 150 µg PDL-1 antibodies per treatment, were given 3 days apart starting from 3rd day of B16F10 inoculation. Tumor growth was monitored daily, and mice were sacrificed on day 16 for comprehensive analysis of tumor tissues and serum cytokines.

#### *Ex vivo* splenocyte stimulation assay

Spleen tissue collected from B16F10 and CT26 tumor bearing mice were passed through 70μm cell strainer to get single cell suspension. The cells were then washed with RPMI media and treated with RBC lysis buffer for 10 min at room temperature, followed by two additional washes with RPMI media. Trypan blue exclusion dye was used to assess cell viability. Next, 1 million live cells from each mouse were incubated with 5 μg/mL TRP-2 peptide for B16F10-bearing mice and AH1 peptide for CT26-bearing mice at 37°C with 5% CO₂ in the presence of 3 μg/mL Brefeldin A. After 24 h, suspension cells were collected from the culture media via centrifugation and analyzed for T cell activation using flow cytometry.

#### Flow cytometry analysis

For the in vitro BMDC stimulation assay, BMDCs were detached from culture plates using the non-enzymatic dissociation reagent Versene by incubating for 10 min at 37 °C. Cells were then washed twice with PBS and resuspended in FACS buffer (PBS with 2% FBS) for staining.

Single-cell suspensions were prepared from tumors, TDLNs, and spleens. Tumor tissues were enzymatically digested using Liberase TM (200 U/mL) and DNase I (50 μg/mL) for 30 min at 37 °C, followed by filtration through a 70 μm cell strainer (Corning Inc., Corning, NY, USA). Cells were washed twice with PBS and treated with RBC lysis buffer for 20 min at room temperature followed by 2 washed with FACS buffer. Single cell suspensions were incubated with Live/Dead stain following manufacturer’s protocol and then stained 60 min on ice in dark with specific anti-mouse fluorophore-conjugated antibodies for surface marker staining. For intracellular cytokine staining, cells were fixed and permeabilized using a BD Transcription Factor Buffer Set and stained for CD206, FOXP3, IFNγ, IL-2, and Granzyme B for 60 min in the dark on ice. Data were acquired using BD LSR II or Agilent Novocyte 3000 flow cytometer and analyzed using FlowJo software v. 10.2 (TreeStar; RRID:SCR_008520) or NovoExpress software v1.6.2. Compensation and gating strategies were validated with unstained and single-stained controls.

Gating for different immune cell population were done as follows: CD45^+^ (Tumor infiltrating leukocytes; TILs), CD45^+^ CD3^+^ (Total T cells), CD45^+^ CD3^+^ CD4^+^ CD8^-^ (TH, CD4^+^ T helper cells), CD45^+^ CD3^+^ CD8^-^ CD4^+^ FOXP3^+^ (Treg, Regulatory T cells), CD45^+^ CD3^+^ CD4^-^ CD8^+^ (TC, CD8^+^ T cells), CD45^+^ CD3^+^ CD4^-^ CD8^+^ GZMB^+^/IFNγ^+^/IL-2^+^ (Activated cytotoxic T-cells), CD45^+^ CD3^+^ CD4^-^ CD8^+^ PD1^+^ (Exhausted T cell), CD45^+^ CD3^+^ CD4^-^ CD8^+^ CD44^-^ CD62L^+^ (Naïve CD8 T cells), CD45^+^ CD3^+^ CD4^-^ CD8^+^ CD44^+^ CD62L^-^ (Effector memory CD8 T cells), CD45^+^ CD3^+^ CD4^-^ CD8^+^ CD44^+^ CD62L^+^ (Central memory CD8 T cells), CD45^+^ CD11b^-^ CD11c^+^ CD8^+^ SIRPα^-^ (cDC1, classical dendritic cell type 1), CD45^+^ CD11b^+^ CD11c^+^ CD8^-^ SIRPα^+^ (cDC2, classical dendritic cell type 2) 46, CD45^+^ CD11b^-^ CD11c^+^ CD8^+^ SIRPα^-^ MHC-II^+^ CD86^+^ (Activated cDC1), CD45^+^ CD11b^+^ CD11c^+^ CD8^-^ SIRPα^+^ MHC-II^+^ CD86^+^ (Activated cDC2), CD45^+^ CD11c^-^ CD11b^+^ (Total Macrophages), CD45^+^ CD11c^-^ CD11b^+^ CD86^+^ MHC-II^+^ (Activated M1 Macrophages), CD45^+^ CD11c^-^ CD11b^+^ CD206^+^ (M2 Macrophages), CD45^+^ CD11b^+^ Ly6C^Hi^ Ly6G^-^ (Monocytic-Myeloid derived suppressor cells, M-MDSC) & Ly6C^Lo^ Ly6G^+^ (Polymorphonuclear-MDSC, PMN-MDSC).

#### Serum cytokine analysis

Blood was collected intracardially on the day of sacrifice of different studies and allowed to clot for 30 min at room temperature. Serum was separated by centrifugation at 2,000 g for 10 min and stored at -80 °C until analysis. Concentrations of cytokines were measured using a multiplex Luminex assay at Eve Technologies Corp. (Calgary, Alberta). The analysis employed the Mouse cytokine/chemokine 32-plex Luminex assay, which assessed 32 analytes. Samples were analyzed on a Luminex™ 200 system, and standard curves were generated for each cytokine to ensure quantification accuracy by Eve Technologies and results were shared in picograms per milliliter concentration.

#### Histology (H&E) analysis

Tumors were harvested 7 days after HT treatment for histological confirmation of treatment effects. Excised tumors were fixed in 10% neutral buffered formalin (at least 24 h), transferred to 70% ethanol, and submitted to the Oklahoma Animal Disease Diagnostic Laboratory (OADDL) at Oklahoma State University, for routine processing. Whole-slide images were acquired by OADDL. H&E sections were reviewed by veterinary pathologist to verify histological evidence consistent with HT treatment (i.e., treatment-region tissue fractionation/necrosis), and representative image is shown in the figure.

#### Statistical analysis

Statistical analyses were performed using GraphPad Prism 10.0 software. Data were expressed as mean ± SEM, and comparisons between two groups were conducted using unpaired t-tests. One-way or two-way ANOVA was applied for multiple group comparisons, followed by Tukey’s or Fisher’s LSD tests as appropriate. Survival curves were analyzed using Kaplan-Meier estimates, and significance was determined using the log-rank test. P values less than 0.05 were considered statistically significant and are denoted in figures as */# p < 0.05, **/## p < 0.005, ***/### p < 0.0005, ****/#### p < 0.0001.

## Results

### Microparticles loading and release characterization

**Figure 1:**
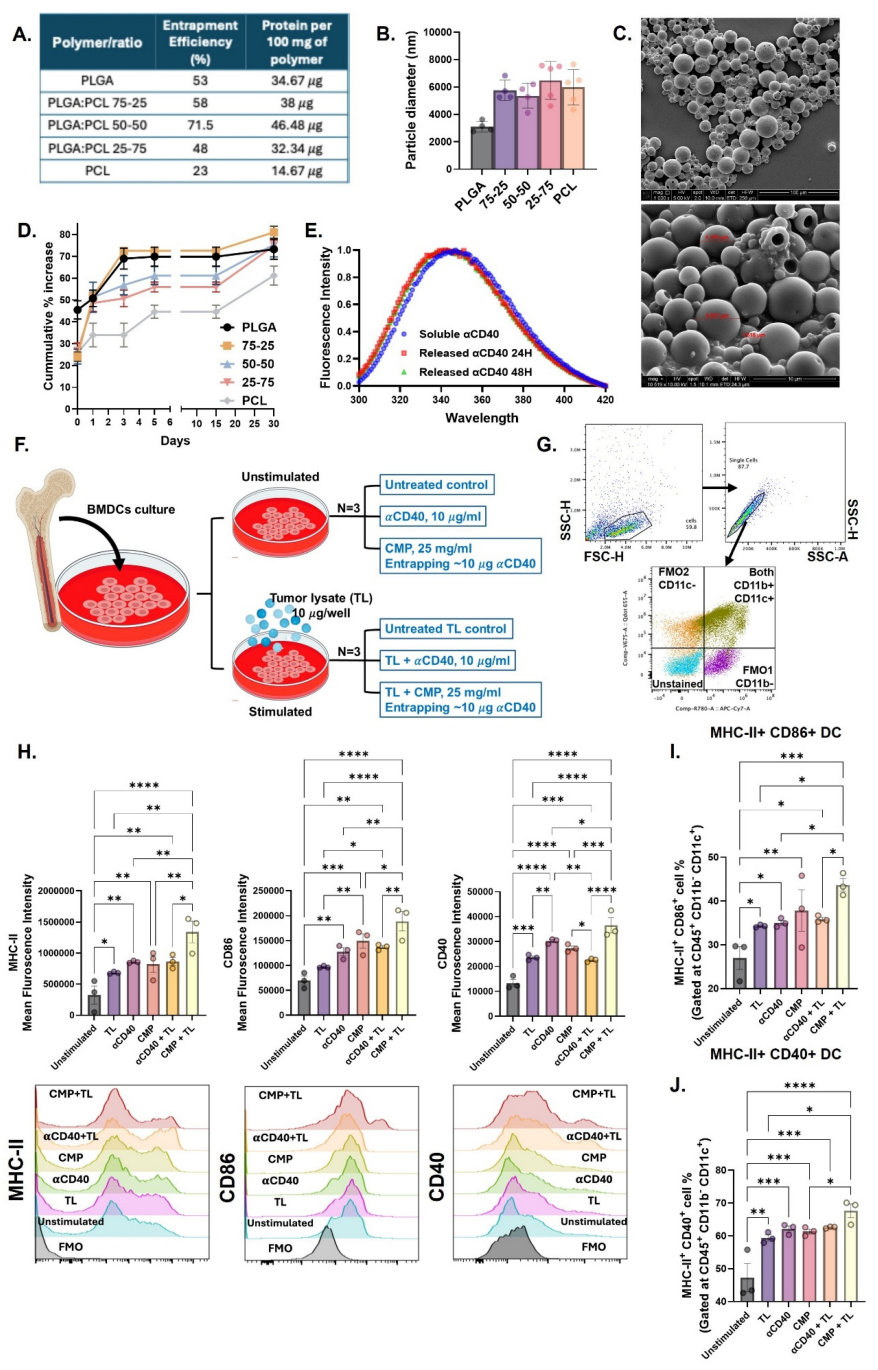
** Characterization and immunological assessment of αCD40 antibody encapsulated microparticles (CMPs). A)** Loading realization % of Microparticles (MPs) prepared using different ratios of PLGA & PCL using IgG antibody (150kD) as model protein. **B)** Hydrodynamic diameter (in μm) of MPs determined using dynamic light scattering (DLS) analysis (n = 5). **C)** Scanning electron microscopy images of CMPs (100 μm and 10 μm scale). **D)** Cumulative percent release of loaded IgG from MPs over 30 days (n = 5). **E)** Intrinsic protein fluorescence spectra of αCD40 antibody released from CMPs after resuspension in PBS for 24 h (red) and 48 h (green), compared to αCD40 diluted in PBS (blue). **F-G)** Schematic of BMDC stimulation assay with CMP and tumor-associated antigens (B16F10 tumor cell lysate) & gating strategy employed. **H)** Mean fluorescence intensity (MFI) of activation markers MHC-II, CD40 and CD86 on the surface of DCs post stimulation (Gated on CD11c; n = 3). **I)** Frequency of MHC-II^+^ CD86^+^ double positive DCs (% of CD11c^+^; n = 3). **J)** Percentage of MHC-II^+^ and CD40^+^ double positive DCs (% of CD11c^+^; n = 3). Statistical analysis: One way ANOVA followed by Tukey Test used for immune cell analysis. * p < 0.05, ** p < 0.005, *** p < 0.0005, **** p < 0.0001.

To support sustained αCD40 availability within the TME for prolonged APC receptor engagement, we developed PLGA/PCL composite microparticles (MPs) rather than nanoparticles that are rapidly phagocytosed by APCs. MPs limit rapid particle internalization, favoring extracellular and local presentation, and thereby reduce reliance on APC endocytosis and lysosomal trafficking 47, 48. MPs made solely from PCL or PLGA achieved loading efficiencies between 25-50%. In contrast, MPs synthesized using a 50:50 PLGA-PCL blend demonstrated a significantly higher loading ~72%, corresponding to ~46.5 µg of IgG per 100 mg of MPs (Figure 1A). The incorporation of PCL with relatively higher molecular weight and lower degradation rate than PLGA increased the average MP diameter from ~3 µm to ~6 µm, irrespective of PCL concentration (Figure 1B). This was evidenced in SEM imaging that confirmed the spherical morphology and size of the PLGA-PCL MPs (Figure 1C). Next, the release kinetics of the encapsulated protein were examined. PLGA MPs exhibited a burst release, with over 45% of the IgG antibody released within the first 2 h (Figure 1D). In contrast, the inclusion of PCL with lower degradation rate in PLGA reduced the initial release to 20-25% from MPs, extending the release period to 6 days. In fact, MPs composed entirely of PCL demonstrated the slowest release profile.

### CMPs activated BMDCs in the presence of tumor lysate

Based on above findings, the PLGA-PCL (50:50) MPs were selected for encapsulating the αCD40 antibody. We tested if the αCD40 antibody encapsulated within 50:50 PLGA-PCL MPs (CMP) maintained its structural integrity. This was verified by spectrophotometric assessment of tertiary conformation by tryptophan emission spectra, wherein the encapsulated αCD40 fluorescence emission patterns were comparable to those of the unencapsulated antibody (Figure 1E).

Further, we assessed the functional activity of encapsulated αCD40 by incubating CMP with harvested BMDC in combination with tumor-associated antigen stimulation (Figure 1F) by examining increased surface expression of commonly studied DC activation markers like MHC-II, CD40, and CD86. Tumor-associated antigens provide a specific stimulus that mimics the presence of tumor cells, creating a relevant scenario to test how αCD40 antibodies enhance the DCs ability to recognize and present antigens, which is crucial for initiating a tumor-specific immune response. Data suggested that CD11b^-^ CD11c^+^ BMDCs (Figure 1G) stimulated with CMP in the presence of B16F10 tumor cell lysate (TL) exhibited increased expression of activation markers MHC-II, CD40, and CD86 (Figure 1H, 1I & 1J), compared to αCD40 alone. Moreover, CMP stimulation in the presence of tumor lysate was associated with enhanced MHC-I expression along with CD86 & CD40 (Figure S1). Together, these results indicate that CMPs preserve αCD40 integrity, sustain its bioactivity, and potentiate antigen-driven immune activation within the TME.

### CMP monotherapy outperformed αCD40 in efficacy and immunomodulation

**Figure 2:**
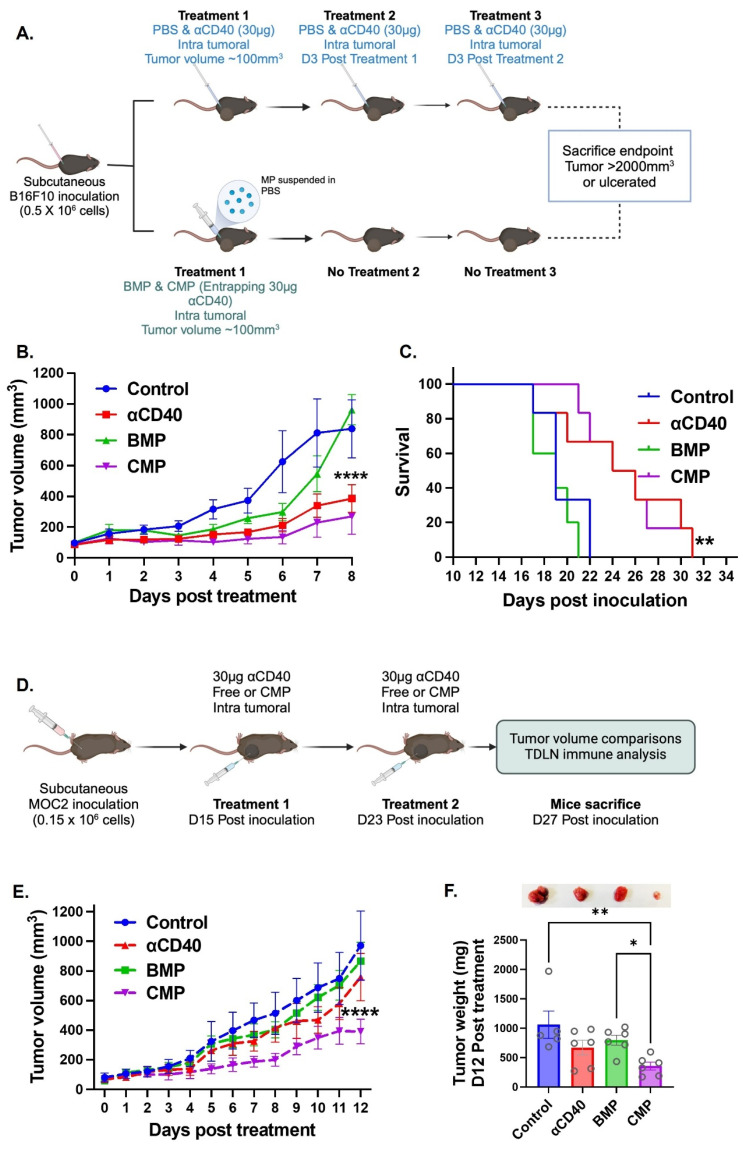
** CMP delivery reduces dosing frequency while maintaining and enhancing αCD40-mediated anti-tumor immunity compared to αCD40 alone in B16F10 and MOC2 tumors. A)** Treatment involved either three weekly intratumoral doses of 30 µg free αCD40 or a single intratumoral dose of CMP containing 30 µg aCD40 in B16F10 mouse melanoma model. **B)** Tumor growth inhibition in the B16F10 model (n = 6). **C)** Survival curve following Kaplan Meier statistical test for B16F10 bearing mice. **D)** CMP efficacy was validated in the poorly immunogenic MOC2 tumor model using weekly administration of either CMP or αCD40 at matched (1:1) αCD40-equivalent dosing. **E)** MOC2 tumor growth inhibition curve (n = 5-6). **F)** MOC2 tumor weights extracted 12 days post treatment from mice (n = 5-6) with representative tumor pictures from each treatment group. Statistical analysis: Tumor growth curves were analyzed by two-way ANOVA with Tukey’s test. Tumor weights were analyzed by one-way ANOVA with Tukey’s test. Survival curves were compared using the log-rank (Mantel–Cox) test. *p < 0.05, **p < 0.005, ***p < 0.0005, ****p < 0.0001.

A key goal of developing CMPs was to sustain APC activity in TME through timed release of encapsulated αCD40. We therefore compared single CMP administration with three bolus intratumoral αCD40 doses in B16F10 tumors (Figure 2A). Both treatments produced comparable tumor growth inhibition (Figure 2B) and survival outcomes (Figure 2C), with CMPs showing relatively greater efficacy despite requiring only one-third the dosing frequency. The enhanced efficacy of CMP was also reproducible in the poorly immunogenic MOC2 tumor model. In this setting, we reduced the αCD40 dosing frequency to match the once-weekly CMP regimen (Figure 2D). Under 1:1 (αCD40: CMP) matched dosing condition, CMP efficacy was maintained, whereas αCD40 activity declined. For example, weekly CMP administration led to a 2.7-fold reduction in MOC2 tumor growth, while free αCD40 and isotype antibody–loaded blank microparticles (BMPs) showed no significant effects compared with controls (Figure 2E & 2F). Together, these findings indicate that intratumoral CMPs can achieve superior outcomes compared to repeated αCD40 injections, and their efficacy in both B16F10 and MOC2 tumors underscores the broad, tumor-agnostic potential of this therapeutic strategy (Figure 2B & 2E).

**Figure 3:**
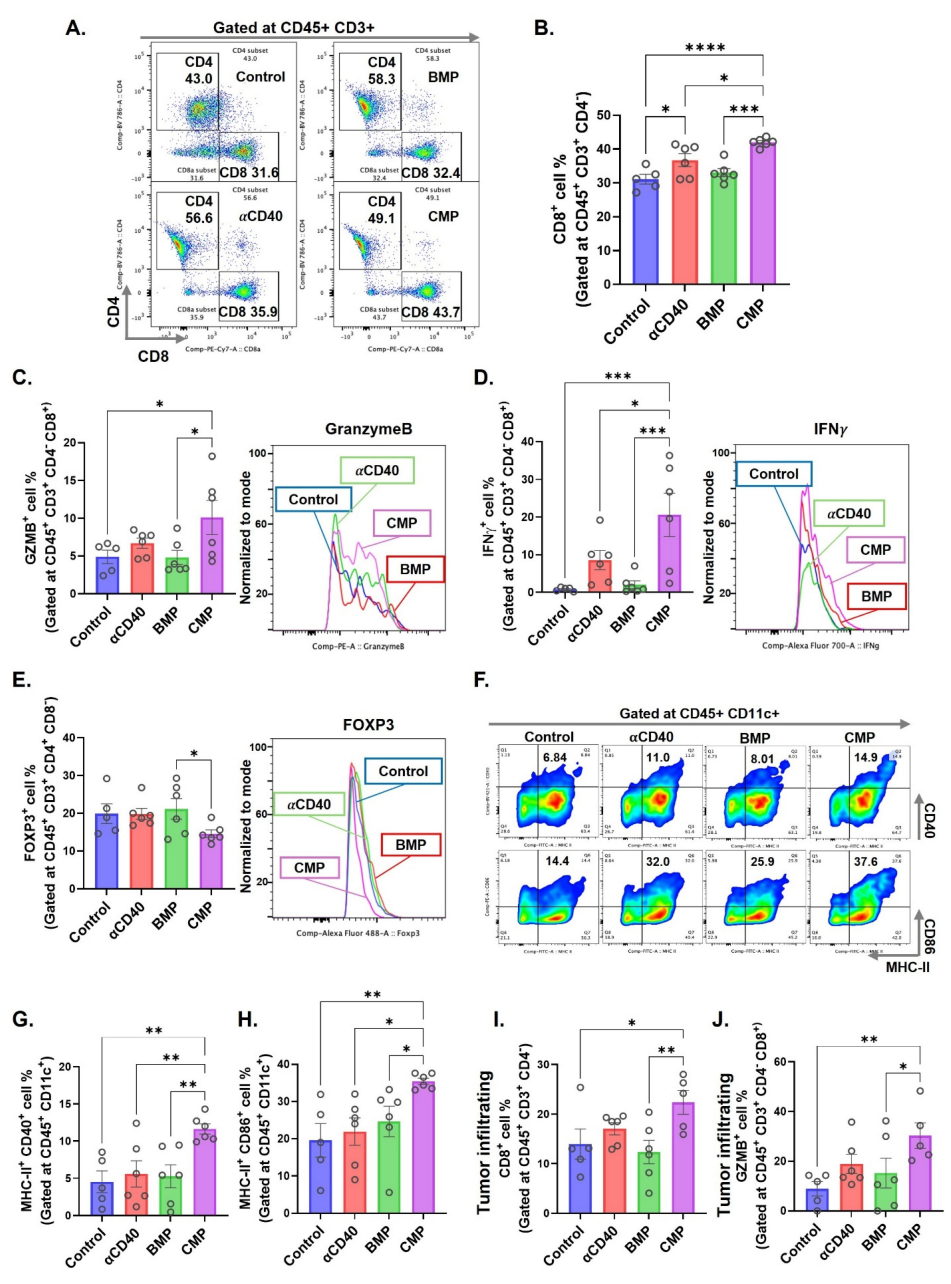
** Intratumoral administration of CMP elicits robust dendritic cell mediated CD8 T cell activation at tumor draining lymph node in MOC2 mouse model. A)** Selection of CD4 & CD8 T cells on CD45^+^ CD3^+^ gated cells accumulated in TDLNs of MOC2 bearing mice from different treatment group (from Figure 2D; n = 5-6). **B)** CD8+ T cells shown as a percentage of total CD3^+^ T cells in TDLNs. **C&D)** Intracellular expression of activation markers, (C) granzymeB (GZMB) and (D) IFNγ represented as positive cell percentage of CD8^+^ T cells present in TDLNs. **E)** Frequency of FOXP3^+^ CD4^+^ regulatory T cells (% of CD4^+^) in TDLNs. **F)** Represented flow cytometry gates for MHC-II and CD40 double positive cells and MHC-II and CD86 double positive cells gated at CD11b^-^ CD11c^+^ DCs in TDLNs of different treatment groups. **G)** Frequency (% of CD11c^+^) of MHC-II^+^ CD40^+^ DCs in TDLNs. **H)** Frequency (% of CD11c^+^) of MHC-II^+^ CD86^+^ DCs in TDLNs. **I)** CD8^+^ T cells infiltrating tumors shown as a percentage of total CD3^+^ T cells (n = 5-6). **J)** Intracellular expression of activation markers, GZMB represented as positive cell percentage of CD8^+^ T cells infiltrating tumors. Statistical analysis: One-way ANOVA with Tukey test was used for immune cell analysis. * p < 0.05, ** p < 0.005, *** p < 0.0005, **** p < 0.0001.

Given that presence of GZMB⁺ CD8⁺ T cells in TDLN reflect the cytotoxic activation, we analyzed DC and T cell populations in the TDLNs of treated MOC2-bearing mice. αCD40-treated group showed a significant increase in CD8^+^ T cells within the TDLNs, however, this effect was further enhanced in CMP-treated mice compared to free αCD40 (Figure 3A & 3B). Notably, CD8⁺ T cells in the CMP group expressed significantly higher levels of cytotoxic markers, including granzyme B (Figure 3C) and interferon-gamma (IFNγ) (Figure 3D) compared to other treatment groups. In addition, CMP treated mice exhibited fewer Treg cells (CD4⁺ FOXP3⁺) in the TDLNs relative to other groups (Figure 3E). While free αCD40 treatment did not alter activated DC populations compared to controls, CMP-mediated sustained release of αCD40 within the TME promoted the accumulation of activated CD11b^⁻^ DCs (representative flow plots across groups shown in Fig. 3F), specifically MHC-II⁺CD40⁺ (Figure 3G) and MHC-II⁺CD86⁺ (Figure 3H), in the TDLNs. By contrast, no notable changes in CD11b⁺ DCs were observed across treatment groups (Figure S2). This persistent presence of activated DCs in TDLNs likely contributed to the enhanced activation of anti-tumor CD8⁺ T cells within tumors (Figure 3I & 3J). However, the tumors eventually progressed, prompting us to compare its efficacy as a monotherapy versus antigen-stimulated HT, as described below.

### CMPs harness HT-derived tumor proteins including damage-associated molecular patterns (DAMPs) to enhance vaccine immunity

**Figure 4:**
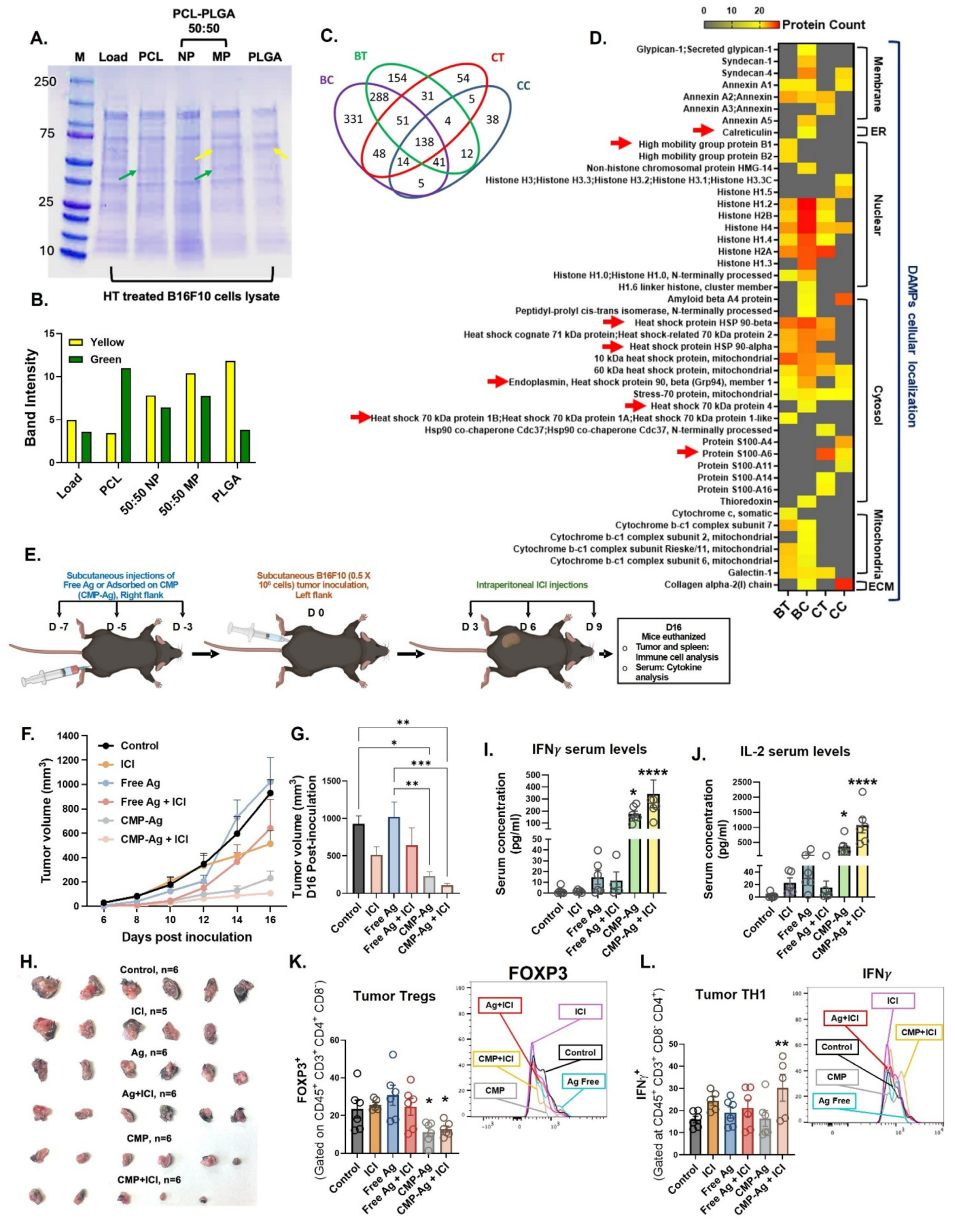
** CMP-mediated capture of HT-released lysate (tumor proteins) delayed B16F10 tumor growth following subcutaneous vaccination. A)** SDS-PAGE confirmed unique protein adsorption on CMP (50:50 PLGA–PCL), from both PCL (green arrow) and PLGA (yellow arrow) polymers. **B)** Densitometry of SDS-PAGE bands showing intensity of yellow (PLGA) and green (PCL). **C)** Venn diagram showing unique and shared protein signatures from B16F10 and CT26 cells adsorbed on MPs quantified by LC-MS/MS. HT-lysed supernatants: BT (B16F10) and CT (CT26); 48 h culture fractions: BC (B16F10) and CC (CT26). **D)** Quantitative analysis of MP surface proteins showing DAMPs from cellular compartments; red arrow indicates ICD-associated DAMPs. **E)** CMP-Ag ((CMP loaded with HT-released B16F10 tumor antigens)) administered s.c. on days 7, 5, 3 before tumor inoculation (day 0); ICI on days 3, 6, and 9. **F)** B16F10 tumor growth in immunized mice. **G)** Statistical differences in Day 16 tumor volume (n = 5/6). **H)** Tumors isolated from different treatment groups on Day 16. **I-J)** Serum levels (pg/mL) of IFNγ (I) and IL-2 (J) cytokines. **K)** Percentage of FOXP3⁺CD4⁺ regulatory T cells (% of CD4⁺ cells, gated on CD45⁺ CD3⁺ CD8⁻) in tumors. **L)** Percentage of activated TH1 cells (IFNγ⁺CD4⁺ T cells; % of CD4⁺ cells) infiltrating tumors. Statistical analysis: Tumor growth curves analyzed using two-way ANOVA with Tukey’s test. One-way ANOVA with Tukey’s test or Fisher’s LSD test used for day 16 tumor volume, immune cell and cytokine analyses. *p < 0.05, **p < 0.005, ***p < 0.0005, ****p < 0.0001.

To determine whether MPs facilitate antigen adsorption, we demonstrated the binding of FITC-conjugated BSA protein to blank 50:50 PLGA-PCL MPs (Figure S3A). Protein adsorption was further optimized by varying fetal bovine serum (FBS) concentrations (1.5–100%) and incubation times (0.5–12 h). Our results showed that 1 mg of MPs could adsorb up to 1 mg of protein within 2 h (Figure S3B), with the adsorbed proteins gradually releasing in phosphate-buffered saline (PBS) over a period of 9 days (Figure S3C & S3D). Given that HT rapidly generates antigen depots, we next assessed whether CMPs could capture HT-released tumor antigens. Lysates from HT-treated B16F10 (melanoma) and CT26 (colon carcinoma) cells (Figure S4A) were incubated with PCL, PLGA, or 50:50 PLGA:PCL microparticles. Protein adsorption varied with polymer type. SDS-PAGE analysis showed that CMPs formulated with 50:50 PLGA-PCL adsorbed proteins characteristic of both PCL (green arrow) and PLGA (yellow arrow), thereby broadening the diversity of proteins interacting with the MPs (Figure 4A & 4B).

Further identification of the tumor proteins adsorbed onto the particles using LC-MS/MS analysis indicated that protein adsorption and enrichment by CMP formulation occurred independently of tumor type (Figure 4C). Importantly, proteins from B16F10 and CT26 cell lysates adsorbed on MPs included DAMPs like calreticulin, HSP-70, HSP-90, HMGB1 associated with immunological cell death (ICD; Figure 4D, red arrow) along with many other DAMPs derived from the cell membrane, endoplasmic reticulum (ER), nucleus, cytosol, mitochondria, and extracellular matrix (ECM). This suggested that CMP formulation effectively captured tumor immune antigens across diverse cellular compartments, potentially making them a promising tool for antigen presentation in cancer immunotherapy (Figure 4C). We next analyzed the synergistic collaboration between CMP-released antigens and αCD40 activation in B16F10-bearing mice. Since CMP or αCD40 alone slowed tumor growth but did not induce complete remission (Figure 2B & 2E), and our goal was to establish antigenic synergism, these monotherapies were not considered as vaccination candidates. CMPs were loaded in vitro with tumor proteins generated from HT-lysed B16F10 cells and tested as a prophylactic vaccination approach, followed by post-challenge ICI to assess antigenic synergy (Figure 4E). Subcutaneous injection of antigen-adsorbed CMPs (CMP-Ag) significantly delayed tumor growth compared to antigen injection alone, with the combined CMP-Ag + ICI treatment demonstrating the most pronounced therapeutic efficacy (Figure 4F, 4G & 4H). CMP-Ag treated mice had significantly higher levels of circulating IFNγ (Figure 4I) and IL-2 (Figure 4J) in serum. Along with these, we observed increased levels of IL-9, CXCL-1, CCL-3, IL-13, CCL4 with CMP-Ag treatment (Figure S4B). Compared to other treatment groups, CMP-Ag had decreased levels of IL-10. CMP-Ag + ICI mice also had increased serum levels of GM-CSF, IL-5, IL-9, IL-15, IL-17, MCP-1, MIG, RANTES and VEGF (Figure S4B). These findings suggest that subcutaneous priming with CMP-Ag vaccine and ICI induced a robust cytokine response by enhancing antigen presentation and APC activation (Figure S5A & S5B), facilitated by sustained delivery of tumor antigens and CD40 agonist compared to untreated control. CD11b^+^ DCs did not show similar activation with any of the treatment (Figure S5C). The activation of CD11b^-^ DCs promoted T cell priming, leading to the secretion of pro-inflammatory cytokines such as IFNγ, IL-2, and GM-CSF, which amplify immune cell proliferation and cytotoxic activity (Figure S5D). Increased chemokines like CXCL-1 and CCL-3 further recruited immune effector cells, while reduced IL-10 levels alleviated immunosuppression within the TME evident by decrease FOXP3 expression and increase in IFNγ on CD4^+^ T cells (Figure 4K & 4L). The cytokine network stimulated by CMP-Ag generated systemic immune responses, enhancing immune cell trafficking, collectively driving anti-tumor immunity.

### HT locally boosted CMP efficacy in poorly & moderately immunogenic melanoma and colon tumors, and improved ICI efficacy

**Figure 5:**
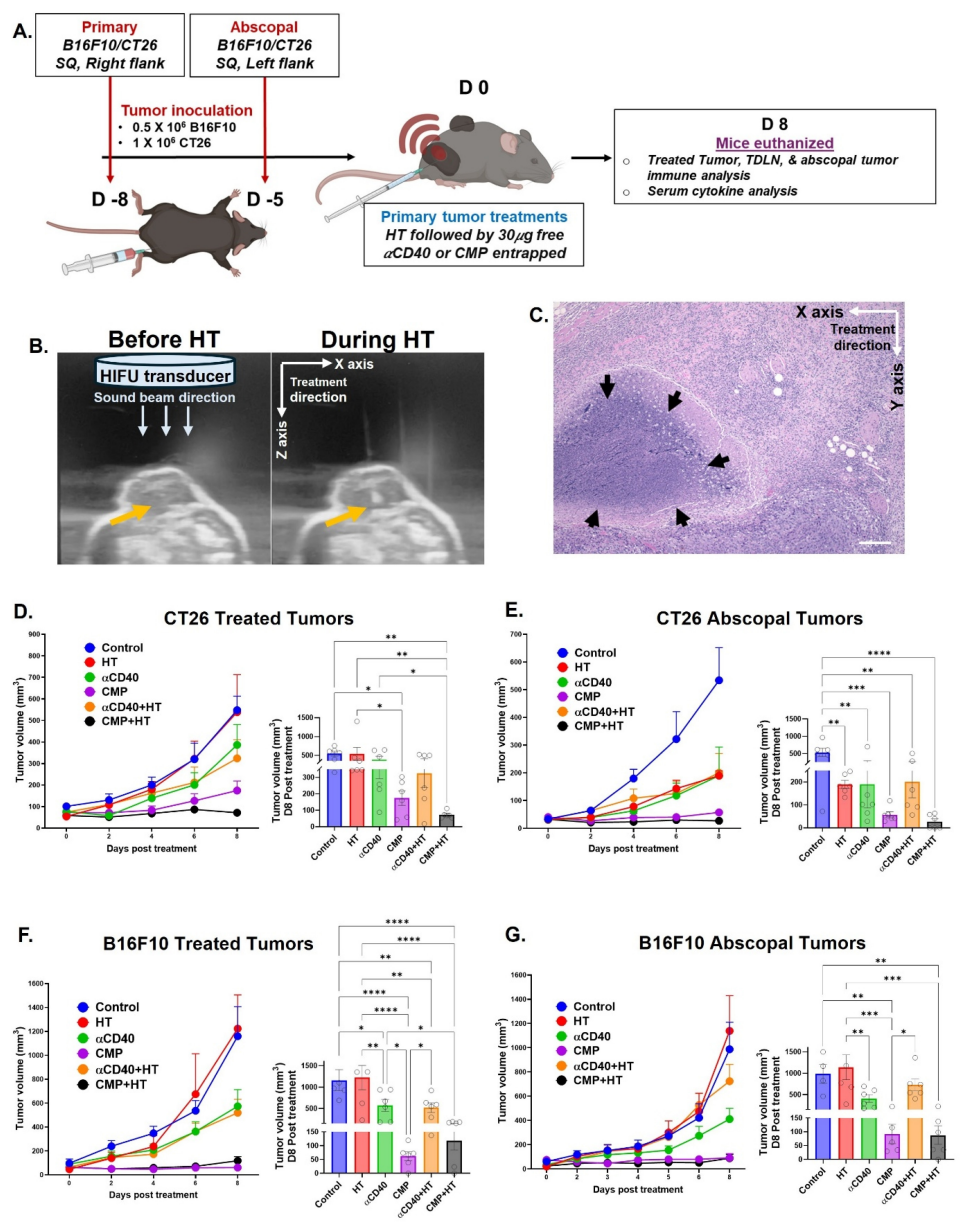
** Intratumoral CMP therapy sustains both local and abscopal tumor growth control in poorly and moderately immunogenic tumor models. A)** Experimental timeline of the bilateral subcutaneous model used to evaluate local and abscopal effects of HT (~10% tumor volume treated) with CMP in moderately immunogenic CT26 (colon carcinoma) and poorly immunogenic B16F10 (melanoma) tumors, n = 6. **B)** B-mode ultrasound image showing hyperechogenicity during HT exposure, confirming bubble formation.** C)** H&E-stained tumor section (10X, 100 μm) showing a defined region of HT-mediated tumor lysis (arrow). **D)** Growth of treated CT26 tumors after HT and αCD40 either delivered in PBS or entrapped in CMP, with statistical analysis of day 8 tumor volume post-treatment. **E)** Growth of untreated abscopal CT26 tumors with statistical analysis of day 8 tumor volume post-treatment. **F)** Growth of treated B16F10 tumors after HT and αCD40 either delivered in PBS or entrapped in CMP, with statistical analysis of day 8 tumor volume post-treatment. **G)** Growth of untreated abscopal B16F10 tumors with statistical analysis of day 8 tumor volume post-treatment. Tumor growth curves were analyzed by two-way ANOVA with Tukey’s test. Tumor volumes on day 8 were analyzed by one-way ANOVA with Tukey’s test. *p < 0.05, **p < 0.005, ***p < 0.0005, ****p < 0.0001.

To assess whether local HT+CMP treatment elicits systemic antitumor responses, we employed a bilateral tumor design using B16F10 and CT26 models. Mice bearing a treated primary flank tumor and a contralateral untreated secondary tumor (Figure 5A) were monitored to determine whether local therapy could restrain growth at the distant untreated site. A primary tumor was implanted on one while a secondary tumor was implanted on the opposite flank 3 days after the primary to serve as a smaller, untreated ‘metastatic’ site. This staggered design accounted for the aggressiveness of the models, allowing 1–2 weeks of post-treatment monitoring. In untreated controls, tumor burden from the chosen inoculum dose might have led to rapid morbidity, precluding evaluation of immune responses. The delayed secondary inoculation also ensured sufficient time for adaptive immunity to develop and for primed immune cells to traffic to distant tumor sites, which would have been missed with simultaneous implantation. Boiling HT was delivered using a 1.5 MHz transducer (Alpinion VIFU2000) at 5 Hz PRF, 1% duty cycle (2 ms on/198 ms off), and 600 W input power. Each focal spot received a 20 s treatment time per focal point, producing an acoustic focus of ~1 mm × 10 mm. Treatments were rastered for low to high coverage of tumor volume (~10% low HT; >50% high HT). HT-induced lysis was confirmed by H&E staining (Figure 5B & 5C). While combination treatments with high-volume ablation (>50%) elicited immunomodulatory changes and tumor efficacy, combining CMPs with low-volume ablation similarly generated robust and sustained anti-tumor immune response, likely due to better preservation of peritumor vasculature and architecture. Thus, data from high ablation experiments are provided in the Supplementary section (Figure S6–S8), whereas all low HT results are described in the following sections.

In CT26-bearing mice, HT alone didn’t affect tumor growth at the treated site (Figure 5D), but significantly reduced tumor growth (~2.5-fold) at the untreated (abscopal) tumor compared to PBS control, similar to the αCD40 control (Figure 5E). In contrast, HT combined with αCD40 decreased growth at both treated and abscopal sites. Notably, CMP+HT was the most effective, reducing treated tumor volume by 7.7-fold vs controls and 2.6-fold vs CMP alone, with 3/6 mice showing no detectable abscopal tumors by day 8 post-treatment. In B16F10-bearing mice, HT didn’t delay tumor growth (Figure 5F & 5G), but αCD40 treatment significantly reduced tumor growth at both the treated and abscopal sites (~2.2-fold compared to control). CMP(±HT) was even more efficacious with ~20-fold at the treated site and ~12.5-fold at the abscopal site.

**Figure 6:**
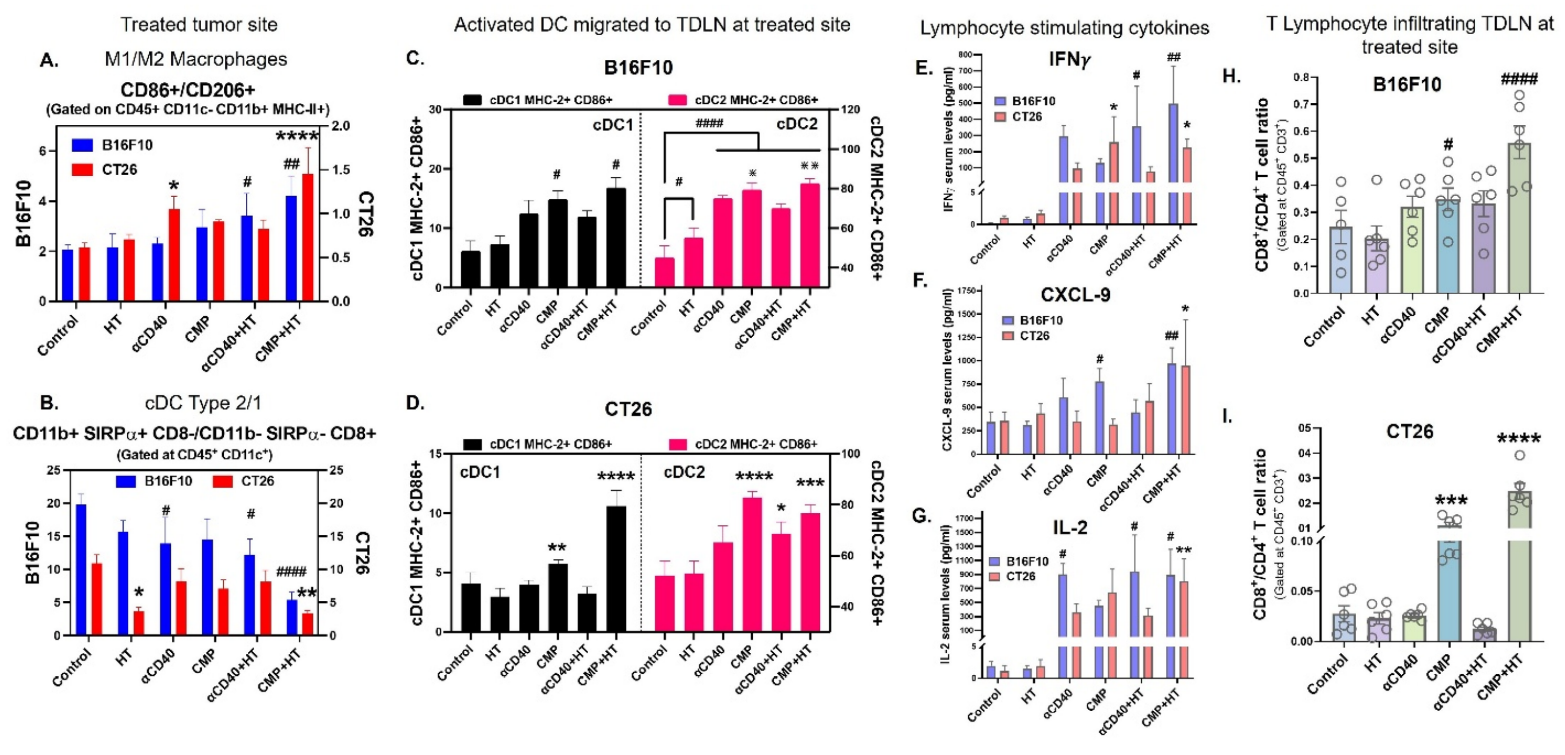
** Sustained release of tumor proteins and αCD40 from CMPs promoted cDC1 maturation and accumulation to TDLNs, enhancing DC activation, lymphocyte chemotaxis, and cytokine responses in B16F10 and CT26 tumor models. A)** Ratio of M1 macrophage (CD86^+^) to M2 macrophages (CD206^+^) numbers in treated tumors analyzed using flow cytometry. Cells were gated at CD45^+^ CD11c^-^ CD11b^+^. Blue bars represent B16F10 tumor (n = 5/6) and red bars represent CT26 tumor (n = 6) model data. **B)** Ratio of cDC2 (CD11b^+^ SIRPα^+^ CD8^-^) to cDC1 (CD11b^-^ SIRPα^-^ CD8^+^) numbers in treated tumors, gated at CD45^+^ CD11c^+^ cells. **C-D)** Frequency of activated CD11c^+^ DCs, MHC-II^+^ CD86^+^ double positive cells (% of parent population, cDC1 or cDC2) in TDLNs of treated tumors. MHC-II CD86 co-expression on cDC1 represented in black bars and on cDC2 in pink bar in B16F10 tumor (n = 5/6; C) and CT26 tumor (n = 6; D) bearing mice. **E-G)** Serum levels in pg/ml of lymphocyte stimulation cytokines, IFNγ (E), CXCL-9 (F), and IL-2 (G) in B16F10 (blue bars; n = 5/6) and CT26 (red bars; n = 6) model. **H-I)** Ratios of CD8^+^ T cells number to CD4^+^ T cells in TDLNs of treated B16F10 (H) and CT26 (I) tumors. Cells were gated at CD45^+^ CD3^+^. Statistical test for changes in immune cells for two tumor models was conducted separately using One-Way ANOVA with Fisher test. Significant changes for B16F10 model are shown as # and in CT26 model as *. Significant change among αCD40, CMP, αCD40+HT, CMP+HT groups in (C) is shown as ✵. */#/✵ p < 0.05, **/##/✵✵ p < 0.005, ***/### p < 0.0005, ****/#### p < 0.0001.

**Figure 7:**
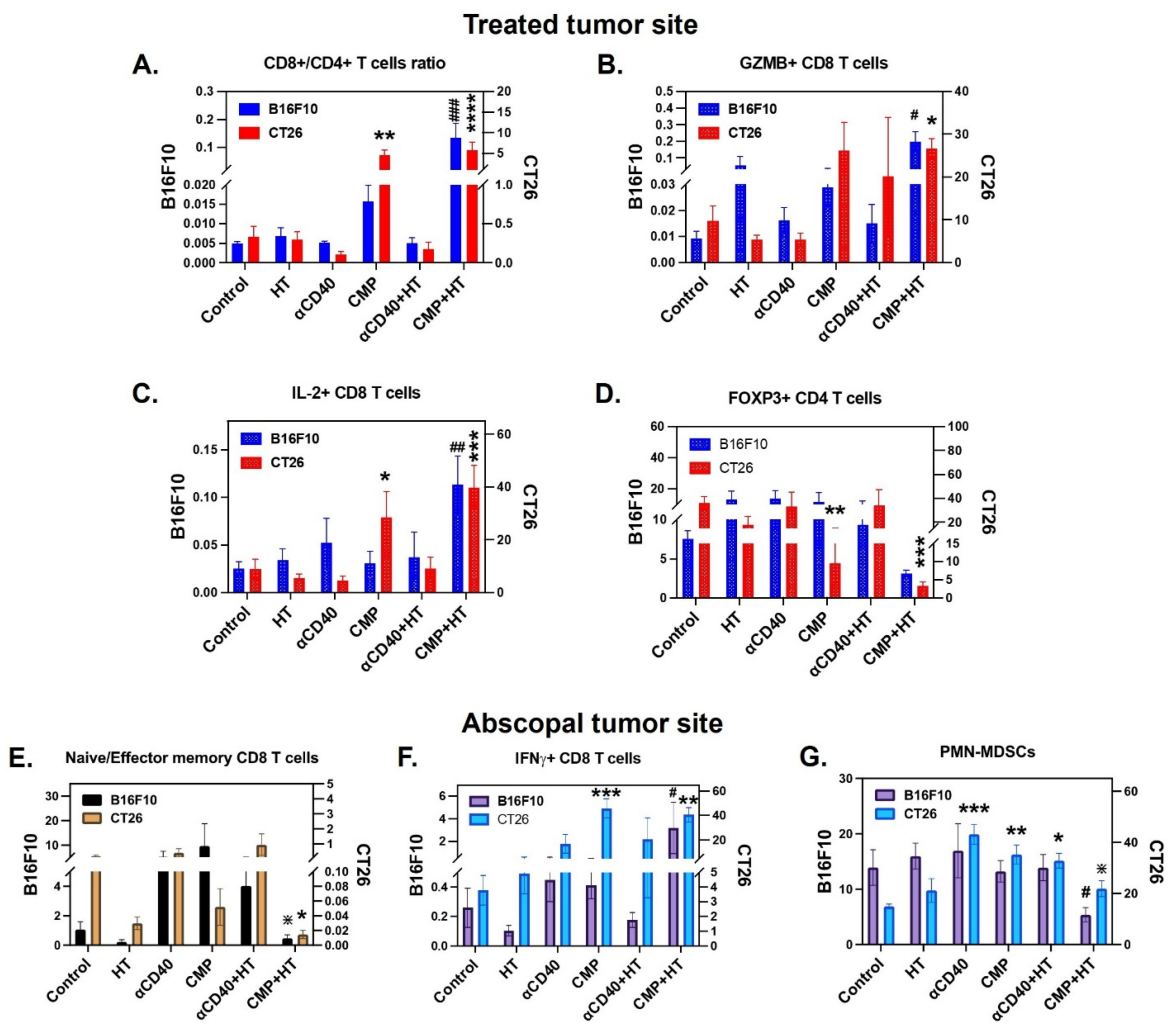
** CMP + HT treatment enhanced systemic cytotoxic T cell activity irrespective of tumor immunogenicity. A)** Ratios of CD8^+^ to CD4^+^ T cells number infiltrating treated B16F10 (blue bars; n = 5/6) and CT26 (red bars; n = 6) tumors. Cells were gated at CD45^+^ CD3^+^. **B-C)** Frequency of cytotoxic marker GZMB+ (B) and activation marker IL-2^+^ (C) CD8^+^ T cells (gated at CD45^+^ CD3^+^ CD4^-^) represented as cells/mg of treated tumor site from B16F10 (blue) and CT26 (red) models. **D)** Frequency of FOXP3^+^ CD4^+^ regulatory T cells (Gated at CD45^+^ CD3^+^ CD8^-^) infiltrating treated tumors of B16F10 (blue) and CT26 (red) models, represented as cells/mg of tumor. **E)** Ratios of Naïve CD44^Lo^ CD62L^Hi^ CD8^+^ T cells to effector CD44^Hi^ CD62L^Lo^ CD8^+^ T cells numbers (Gated at CD45^+^ CD3^+^ CD4^-^) accumulated in untreated abscopal tumors of B16F10 (n = 5/6; black) and CT26 (n = 3/6; yellow) bearing mice. **F)** Frequency of activated IFNγ^+^ CD8^+^ T cells (Gated at CD45^+^ CD3^+^ CD4^-^) infiltrating abscopal tumors of B16F10 (n = 5/6; purple) and CT26 (n = 3-6; blue) tumor model, represented as cells/mg of tumor. **G)** Ly6GHi Ly6C^-^ CD11b^+^ Polymorphonuclear myeloid derived suppressor cell (PMN-MDSC) shown as cells/mg of tumor residing in untreated abscopal tumor of B16F10 (n = 5/6; purple) and CT26 (n = 3-6; blue) tumor model. Statistical analysis: Changes in immune cells for two tumor models were conducted separately using One-Way ANOVA with Fisher test. Significant changes for B16F10 model are shown as # and in CT26 model as *. Significant change among αCD40, CMP, αCD40+HT, CMP+HT groups in (E & G) is shown as ✵. */#/✵ p < 0.05, **/## p < 0.005, ***/### p < 0.0005, ****/#### p < 0.0001.

Immunologically, CMP+HT significantly increased the M1/M2 ratio (Figure 6A), along with M1 & M2 macrophage associated serum cytokine levels (Figure S9A–S9C), and cDC1/cDC2 ratio (Figure 6B) in both B16F10 and CT26 tumors compared to all other treatments at the HT-treated site. This increase was associated with enhanced accumulation of cDC1 and cDC2 to TDLN (Figure 6C & 6D). In CMP+HT treated mice, increased DCs abundance was accompanied with elevated serum levels of lymphocyte stimulating cytokines like IFNγ (Figure 6E), CXCL-9 (Figure 6F) & IL-2 (Figure 6G) in both tumor models. This resulted in increased chemotaxis of CD8 T cells in TDLNs with CMP alone as well as CMP+HT treatments in both B16F10 (Figure 6H) and CT26 (Figure 6I) bearing mice with decreased PD1 expression on these cells (Table S2, Figure S9D). Similar to TDLNs, CD8^+^/CD4^+^ T cell ratio was significantly high with CMP+HT treatments in both tumor models (Figure 7A). Further, CD8 T cells showed significantly higher GZMB (Figure 7B) and IL-2 (Figure 7C) expression & reduced number of Tregs (CD4^+^ FOXP3^+^ T cells, Figure 7D) in both tumor models compared to control. Also, CD8^+^ T cells expressed lower PD1 on their surface with CMP+HT treatment in both tumor models which was otherwise increased with other treatments (Figure S9E).

In untreated abscopal tumors, HT treatment induced higher effector memory (CD44^Hi^ CD62L^Lo^) CD8^+^ T cells in the CT26 and B16F10 tumor models (Figure S9F). Based on these observations, we examined ratio of naïve/effector memory CD8^+^ T cells infiltrating abscopal tumors in various treatment groups (Figure 7E). Among these, CMP+HT treatment lowered naïve/effector memory CD8^+^ T cells ratio relative to other treatments whereas it did not show statistically lower ratio compared to HT alone group. Also, while CD4^+^ T cells didn’t change noticeably relative to control with various treatments (Table S3), CD8^+^ T cells demonstrated increased expression of GZMB and IL-2 with various treatments (Table S3). In addition, CMP+HT treatment significantly increased IFNγ expression on CD8^+^ T cells (Figure 7F), and decreased PMN-MDSCs (CD11b^+^ Ly6C^Lo^ Ly6G^+^) compared to control in the tumors (Figure 7G).

**Figure 8:**
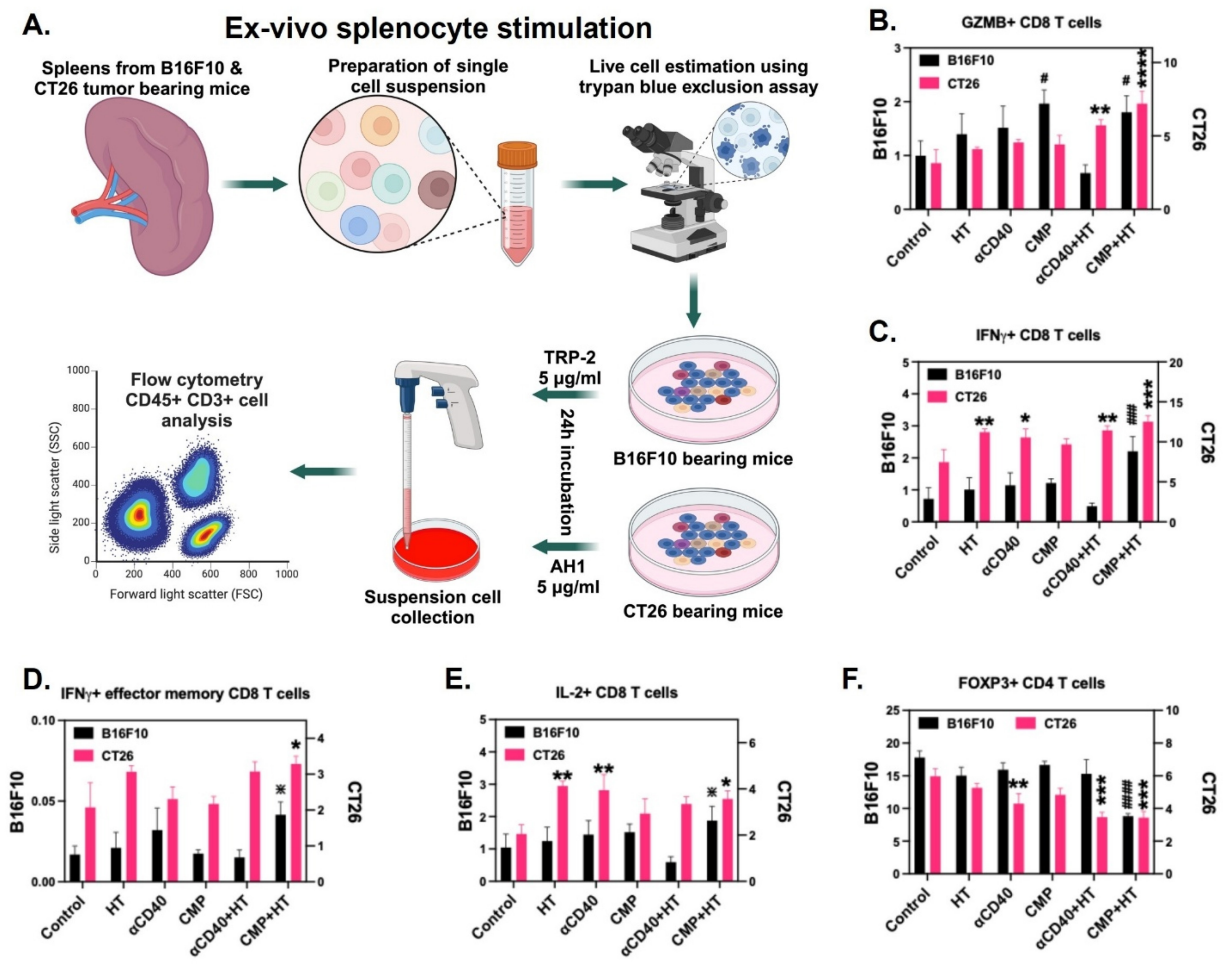
** CMP + HT treatment elicit tumor specific CD8 T cell activation. A)** Experimental plan for stimulating spleen cells isolated from B16F10 & CT26 bearing mice with tumor specific antigens to analyze activation of cytotoxic CD8 T cells. **B)** Frequency of GZMB^+^ CD8^+^ T cells (Gated at CD45^+^ CD3^+^ CD4^-^) represented as % of Total CD3^+^ T cells post-stimulation of splenocytes with B16F10 tumor (n = 5/6; black) specific TRP-2 peptide and CT26 tumor (n = 6; pink) specific AH1 peptide for 24 h. **C)** Frequency of IFNγ^+^ CD8^+^ T cells (gated at CD45^+^ CD3^+^ CD4^-^) shown as % of CD3^+^ total T cells after 24 h stimulation of spleen cells; B16F10-Black, CT26-Pink. **D)** Frequency of IFNγ^+^ effector (CD44^+^ CD62L^LO^) CD8^+^ T cells shown as % of CD3^+^ total T cells from B16F10 (black) and CT26 (pink) tumors bearing mice. **E)** Percentage of IL-2^+^ CD8^+^ T cells (% of CD3^+^ cells); B16F10-Black, CT26-Pink. **F)** Frequency of FOXP3^+^ CD4^+^ Treg cells shown as % of CD3^+^ cells (gated at CD45^+^ CD3^+^ CD8^-^); B16F10-Black, CT26-Pink. Statistical analysis: Changes in immune cells for two tumor models were conducted separately using One-Way ANOVA with Fisher test. Significant changes for B16F10 model are shown as # and in CT26 model as *. Significant change among αCD40, CMP, αCD40+HT, CMP+HT groups in (D & E) is shown as ✵. */#/✵ p < 0.05, **/## p < 0.005, ***/### p < 0.0005, ****/#### p < 0.0001.

Next, to confirm tumor antigen-specific T cell activation, splenocytes isolated from mice in different treatment groups were stimulated ex vivo with TRP-2 peptide for B16F10-bearing mice and AH1 peptide for CT26 tumor-bearing mice (Figure 8A). While all treatment groups exhibited a slight increase in CD8^+^ T cell expression of the cytotoxic marker GZMB post-stimulation, the CMP+HT group demonstrated a significantly greater enhancement in GZMB expression across both tumor models (Figure 8B). IFNγ expression on total CD8^+^ T cells (Figure 8C) as well as CD44^+^ CD62L^Lo^ effector CD8 T cells (Figure 8D) also showed similar trend. Additionally, IL-2 expression in CD8^+^ T cells remained unchanged in B16F10-bearing mice; however, in the CT26 model, an increase in IL-2^+^ CD8^+^ T cells was observed (Figure 8E). Furthermore, CMP+HT-treated mice in both tumor models exhibited a significant reduction in the Treg population among splenic T cells post-stimulation (Figure 8F). HT, αCD40, and HT+αCD40 induced T cell activation in the CT26 model at levels comparable to CMP+HT, despite relatively modest effects on tumor growth with HT alone. This likely reflected the inherently moderate-to-high immunogenicity of CT26 tumors, which can support an activated immune milieu and T cell priming even with limited intervention. In contrast, these effects were not consistently observed in the poorly immunogenic B16F10 model, where CMP+HT elicited significantly greater CD8⁺ T-cell activation, highlighting the importance of combinatorial strategies in overcoming immune resistance in cold tumors.

**Figure 9:**
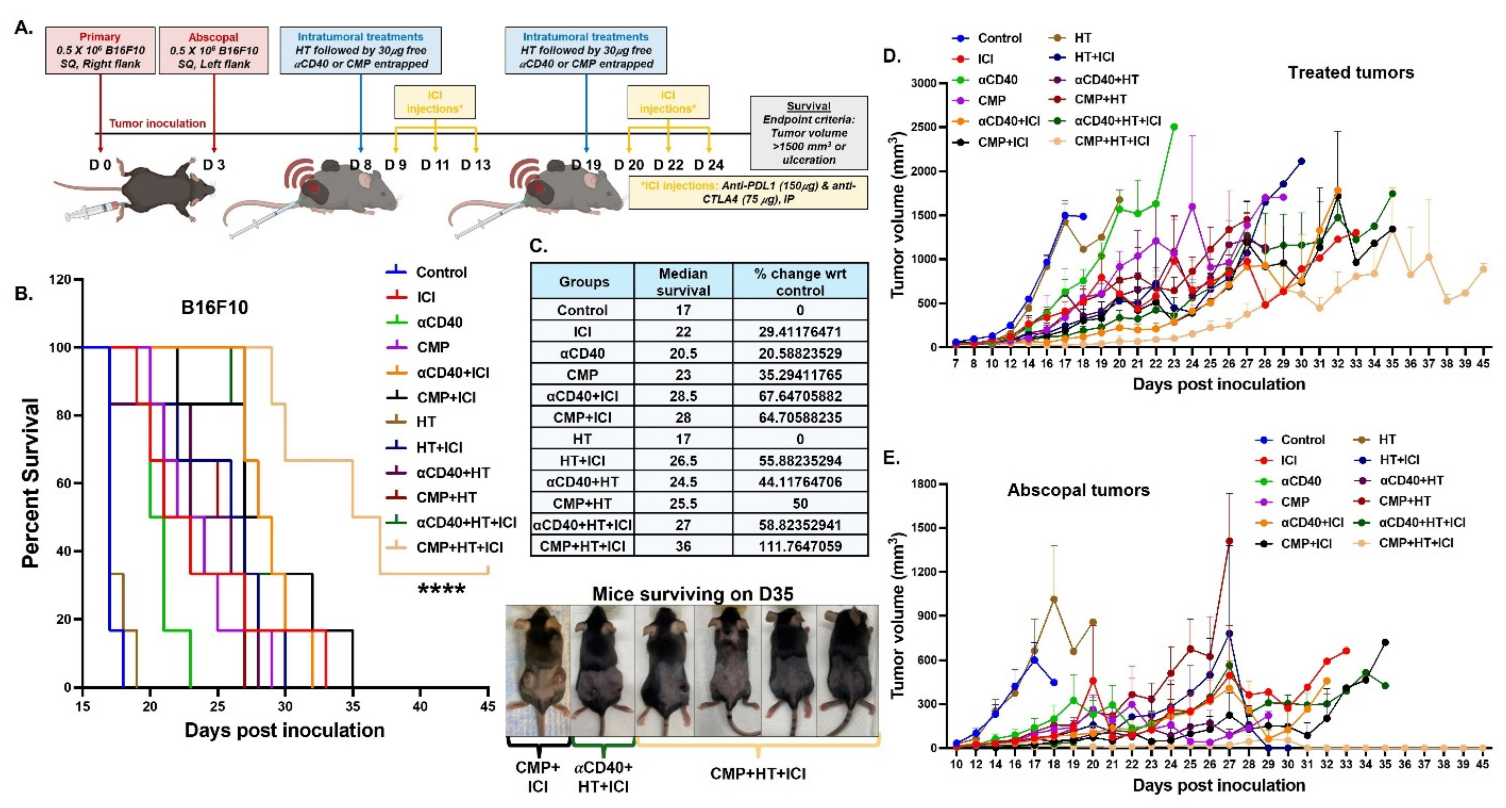
** CMP+HT significantly enhance ICI efficacy and survival of melanoma bearing mice. A)** Treatment timeline showing inoculation of bilateral subcutaneous B16F10 tumors followed by HT and CMP treatment in combination with anti-PDL1 and anti-CTLA4 ICI (n = 6). **B)** Survival curve for B16F10 melanoma bearing mice. **C)** Median survival calculated by Kaplan Meier statistical estimation for each treatment group used to calculate changes in median survival at the baseline of control group using formula ((Test-Control)/Control)*100). Images of mice surviving on day 35 post-inoculation. **D-E)** Line graph representing B16F10 tumor growth at treated site (D) and abscopal site (E). Average tumor volume of each treatment group shown until euthanasia (tumor volume >2500 mm³). Survival curves were compared using the log-rank (Mantel–Cox) test; ****p < 0.0001.

Based on these findings, we evaluated the local and systemic efficacies of CMP and αCD40 in combination with HT and ICIs in B16F10 (Figure 9A) and B16F10-OVA (Figure S10A) model. Overall, HT+CMP was significantly more effective than HT+αCD40 in improving treatment outcomes. In the B16F10 model, 5 out of 6 mice in the CMP+HT+ICI group exhibited abscopal tumor clearance within 16 days of the first CMP+HT treatment. Additionally, while ICI and αCD40 combined with HT improved survival by 30–60% (Figure 9B & 9C), the combination of HT, ICI, and CMP achieved >100% survival vs control due to significant tumor growth retardation at treated and abscopal sites (Figure 9D, 9E & Figure S11). A similar trend was observed in the ovalbumin-expressing B16F10-OVA model, where abscopal tumors cleared in 3 out of 6 mice in the CMP+HT+ICI treatment group within 13 days. Additionally, ICI significantly enhanced median survival, CMP+HT+ICI achieved a 100% improvement relative to the control and nearly doubling survival in the CMP+HT+ICI group compared to the HT+αCD40+ICI group (Figure S10B & S10C).

## Discussion

This study shows that PLGA-PCL-based CMP enables sustained αCD40 delivery and HT-induced antigen release, enhancing APC activation, T cell responses, and synergy with ICIs for superior efficacy. While αCD40 and antigen encapsulation in PLGA carriers has been explored, sustained release with preserved bioactivity remains challenging 49. HT induces immunogenic cell death and antigen depots in the TME 19, 50, but its suppression limits anti-tumor immunity 51. Strategies like systemic adjuvants or bolus antigen delivery aim to stabilize antigens and boost responses but suffer from poor localization and short-lived effects, reducing their efficacy in tumor immunotherapy. By combining FDA approved PLGA and PCL, we optimized αCD40 encapsulation, particle size, and release kinetics, overcoming burst release issues 52. Beyond αCD40, we demonstrate that CMPs efficiently capture and preserve immunogenic proteins like DAMPs, released during HT-induced tumor lysis (Figure 3). This mechanism extends previous work on antigen presentation platforms such as nanoparticles and offers a novel means of synergy with immune checkpoint blockade 53.

The sustained release of αCD40 from CMPs significantly enhanced DC activation, as evidenced by the upregulation of MHC-I, MHC-II, CD40, and CD86. This supports the paradigm that prolonged APC activation is critical for robust CD8^+^ T cell priming and tumor-specific immunity 54. CMPs elicited antitumor immunity in both poorly and moderately immunogenic tumors without requiring tumor-specific neoantigens, thereby simplifying treatment while maintaining efficacy (Figure 5) and minimizing concerns related to precision and non-specific protein adsorption. Scalable manufacturing using microfluidics and particle synthesis can enhance clinical translation. While both low- and high-volume HT induced tumor reprogramming (Figure S8), low-volume ablation combined with CMP therapy interestingly produced sustained antitumor response in our mouse models. This difference likely reflects a dose/coverage-dependent balance between immunogenic tumor disruption and injury-repair programs. HT immune effects have been reported to vary with treatment dose, consistent with a ‘Goldilocks’ regime in which greater tissue destruction does not necessarily yield stronger antitumor immunity 55. In our study, high-volume HT was accompanied by comparatively lower M1/M2 and cDC1/cDC2 ratios, suggesting that more extensive injury may preferentially engage wound-healing myeloid programs that can blunt productive antigen presentation and CD8⁺ priming. Related ablation literature shows that large injury burdens can recruit macrophage-dominated repair responses and immunoregulatory mediators (e.g., TGF-β–associated pathways) that may counteract durable antitumor immunity 56. Conversely, low-volume HT may better preserve peritumoral vasculature and tissue architecture, which can support dendritic-cell trafficking/priming and lymphocyte infiltration, thereby allowing CMP-delivered αCD40 to more effectively amplify APC activation and downstream T cell immunity 57. Given that HT of solid tumors can be time-intensive compared to thermal ablation, the combination of low-volume HT with CMP represents a clinically viable path forward. Intratumoral delivery is clinically feasible for accessible lesions (e.g., superficial tumors or those reachable via image-guided needle placement). However, for deep-seated tumors, lesions near critical structures, or patients with multiple metastatic sites, procedural burden (e.g., anesthesia/sedation, imaging guidance, operator expertise) and risks such as bleeding, infection, pain, or tumor seeding may limit applicability compared with systemic therapies. These limitations could be mitigated by our subcutaneous vaccination strategy that achieved comparable results or by advances in interventional oncology. Future studies in human trials, including investigations on the optimal sequencing of CMP integration, will be essential to optimize outcomes, particularly in solid tumors with dense stromal barriers that resist treatment 58. Overall, this work envisions HT-based multimodal therapies as broadly applicable across tumor types, with advances in image-guided techniques enabling precise intratumoral CMP delivery to further enhance efficacy and improve patient outcomes.

Our vaccination experiments provide proof-of-concept that CMPs loaded ex vivo with tumor lysates generated using HT can elicit systemic immune activation in combination with immune checkpoint inhibition (ICI) (Figure 4). Although our experimental design did not model an autologous, biopsy-derived therapeutic vaccine in a clinical setting and lacked a free antigen + αCD40 control arm which would more directly distinguish the contribution of CMP-mediated antigen delivery from CD40 co-stimulation, our data suggest that this ex vivo antigen-generation approach can produce a broad spectrum of native tumor antigens and DAMPs available for capture by CMPs. These findings support future studies using biopsy- or resection-derived autologous tissue and clinically relevant therapeutic dosing schedules for personalized vaccination.

It also remains unclear whether HT generates a functionally distinct lysate compared with standard mechanical lysis methods (e.g., sonication or homogenization). However, our LC–MS analyses indicate that CMPs can enrich immunogenic proteins, resulting in the most consistent tumor control including abscopal effects and robust dendritic cell/macrophage remodeling with enhanced effector CD8⁺ T cell function across both CT26 and B16F10 models when combined with HT (Figure 3).

Additionally, HT and CMPs resensitized tumors to ICIs, improving survival by reprogramming the immunosuppressive TME. This re-sensitization can be attributed to multiple mechanisms. Poorly immunogenic tumors typically exhibit an immunosuppressive TME that promotes T cell exclusion and resistance to ICI 59. By delivering αCD40 in a sustained manner and capturing antigens released by HT-induced tumor cell lysis following intratumoral immunotherapy, CMPs help reprogrammed the TME, enhancing the functionality of effector T cells. This combination also boosted DC activation, promoted T cell priming in TDLNs, and shifted the microenvironment toward a pro-inflammatory state by reducing Tregs and MDSCs while increasing the M1/M2 macrophage ratio.

The scientific implications of this study extend beyond improving ICI responses. The strong abscopal effects, marked by systemic tumor regression at untreated sites, highlight its potential to induce widespread immune activation (Figure 7). Localized treatment amplified systemic immunity by increasing effector memory T cells and improving activated T cell trafficking to distant tumors (Figure 7E & 7F). ICIs further strengthened this response by blocking inhibitory signals that suppress T cells at untreated sites, consistent with evidence that localized therapies like radiation or thermal ablation enhance systemic immunity when combined with immune modulators 60. These findings support HT, CMPs, and ICIs as an innovative multimodal strategy to overcome resistance in poorly immunogenic tumors, driving durable immunity and improved survival. Future research should explore HT-CMP therapy with immune modulators like STING agonists or TLR ligands to expand its applicability and mechanistic insights. CMPs reduced dosing frequency while maintaining αCD40 efficacy, potentially easing treatment burdens. Although survival data showed no long-term safety concerns, preclinical toxicology studies are needed.

In conclusion, combining HT with CMPs provides a foundation for developing next-generation therapies that improve outcomes for difficult-to-treat cancers. The dramatic improvement in survival rates observed with HT+CMP+ICI treatment can be attributed to multiple synergistic mechanisms: enhanced antigen release and presentation, reprogramming of the TME, restoration of T cell functionality, and induction of systemic immune responses. Together, these findings highlight the potential of combining localized immune-modulatory strategies like CMPs and HT with systemic ICIs to overcome resistance in poorly immunogenic tumors and improve patient outcomes.

## Supplementary Material

Supplementary figures and tables, movie legend.

Supplementary movie 1.

## Figures and Tables

**Table 1:** Composition of IAP and EAP used in microparticle preparation for loading realization % calculations.

Composition of Internal aqueous phase (IAP)	
**Composition**	**Value**
Antibody (αCD40 or isotype)	100 μg
Glycerol	1.25%
PVA	0.63%
Sucrose	10%
pH	7.2
Composition of external aqueous phase (EAP)	
**Composition**	**Value**
CTAB	0.10%
Sucrose	10.00%

## Data Availability

The data generated in this study are available upon request from the corresponding author.
