# Supplementary material for: Converting focused ultrasound–based boiling histotripsy into a systemic cancer vaccine using antigen-capturing microparticles
Source: Theranostics. 2026 May 18;16(12):7007–28. doi: 10.7150/thno.132091 (PMC13232618; doi:10.7150/thno.132091)
Supplement: Supplementary file 1 — Supplementary figures and tables, movie legend. [file thnov16p7007s1.pdf]

Supplementary material for

Converting focused ultrasound–based boiling histotripsy into a systemic cancer vaccine using antigen-capturing microparticles

Akansha Singh *et al.*

*Corresponding author: Ashish Ranjan. Email: ashish.ranjan@utsouthwestern.edu

This PDF file includes:

- Figs. S1 to S11
- Supplementary Table S1 to S3
- Supplementary movie S1

Supplementary Figures:

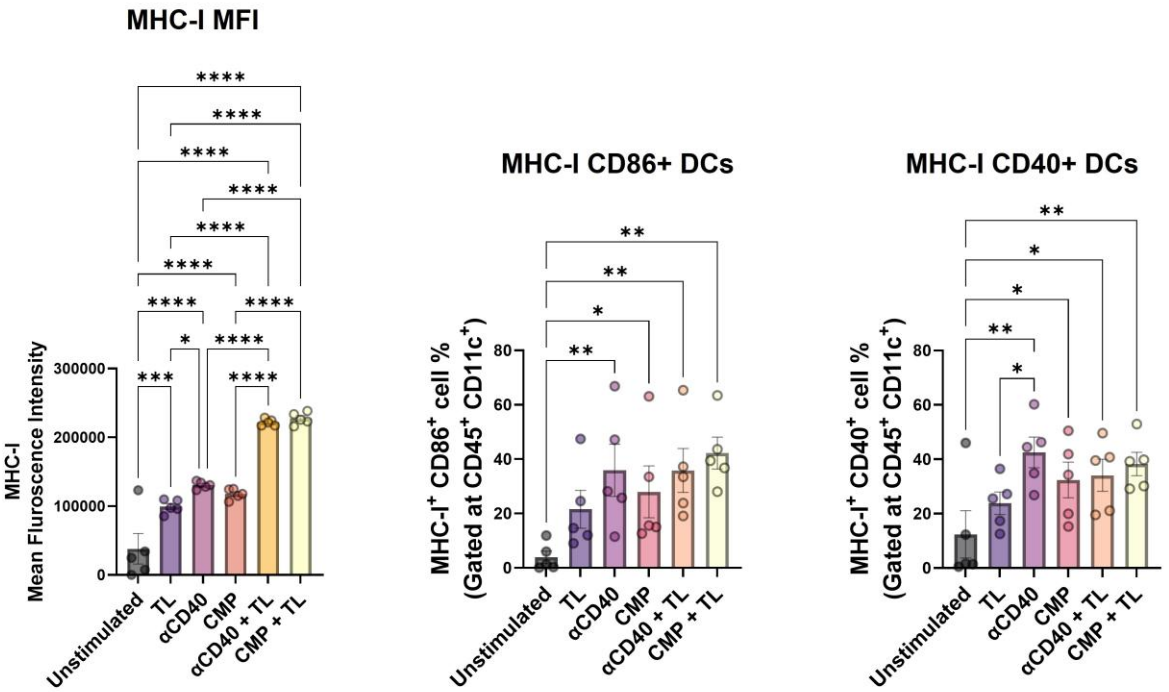

S1. BMDC stimulation assay with CMP and tumor-associated antigens (B16F10 tumor cell lysate). A) Mean fluorescence intensity (MFI) of activation markers MHC-I on the surface of DCs post stimulation (Gated on CD11c; n=5). B) Frequency of MHC-I+ CD86+ double positive DCs (% of CD11c+; n=5). **J)** Percentage of MHC-I+ and CD40+ double positive DCs (% of CD11c+; n=5). Statistical analysis: One way ANOVA followed by Tukey Test used for immune cell analysis. * p<0.05, ** p<0.005, *** p<0.0005, **** p<0.0001.

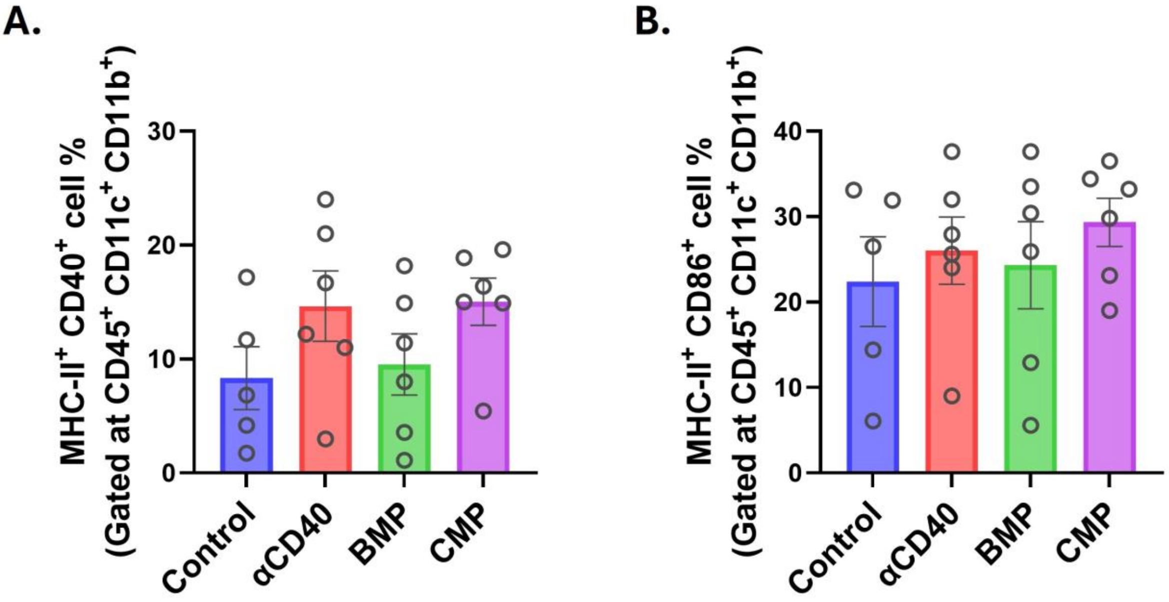

S2. Changes in CD11b+ DCs in tumor draining lymph node (TDLN) post αCD40-microparticle (CMP) treatment in MOC2 bearing mice analyzed using flow cytometry. A) Frequency (% of CD11c+ CD11b+) of MHC-II+ CD40+ DCs in TDLNs. B) Frequency (% of CD11c+) of MHC-II+ CD86+ DCs in TDLNs. Statistical analysis: One-way ANOVA with Tukey test was used for immune cell analysis, no significant change was observed.

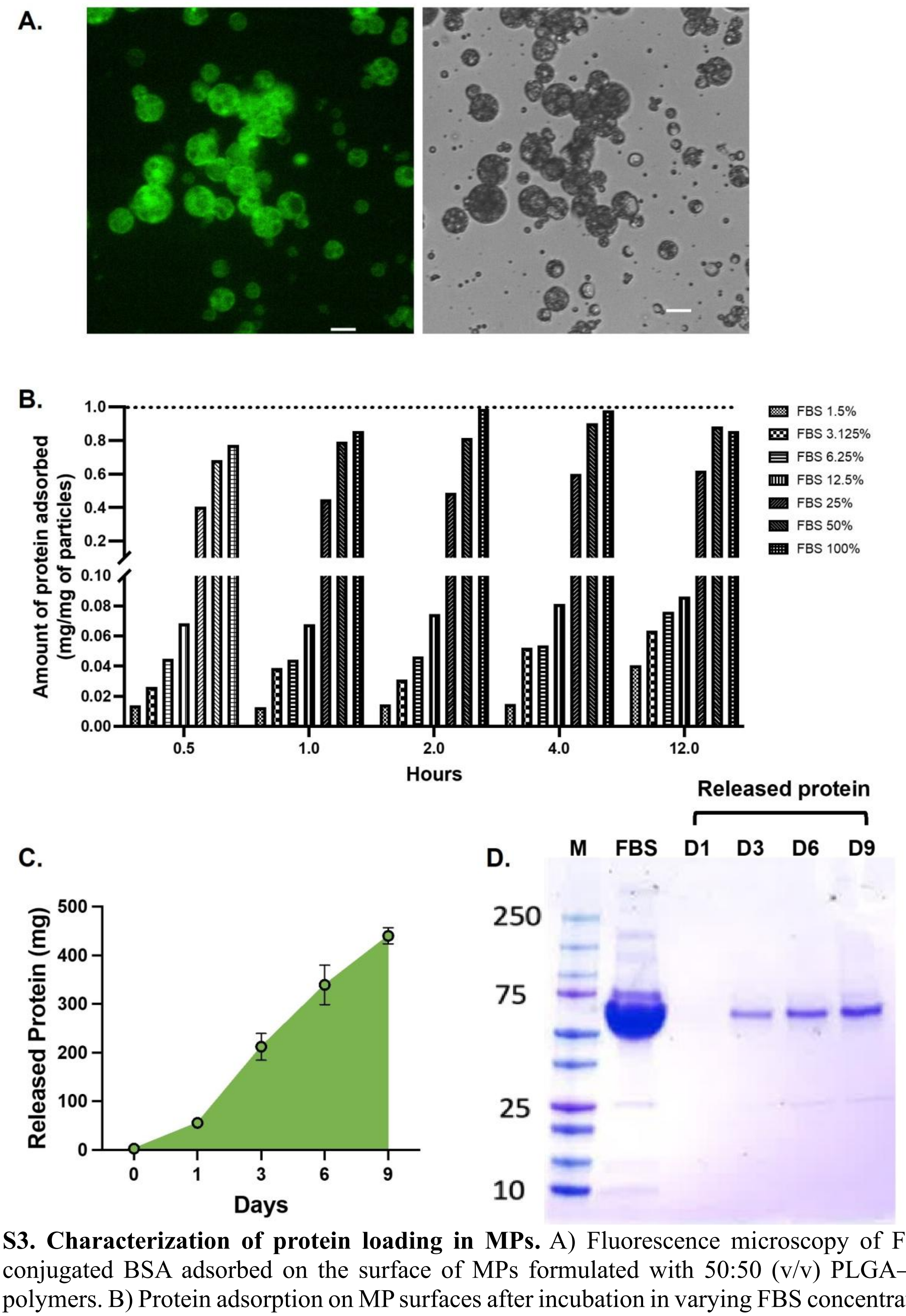

S3. Characterization of protein loading in MPs. A) Fluorescence microscopy of FITC-conjugated BSA adsorbed on the surface of MPs formulated with 50:50 (v/v) PLGA–PCL polymers. B) Protein adsorption on MP surfaces after incubation in varying FBS concentrations (1.5–100%). X-axis: incubation time points (0.5, 1, 2, 4, and 12 h); Y-axis: amount of surface-adsorbed protein. C) Release profile of surface-adsorbed FBS from MPs measured at 1, 3, 6, and 9 days. D) SDS-PAGE gel showing proteins released from MP surfaces at different time points (1, 3, 6, and 9 days).

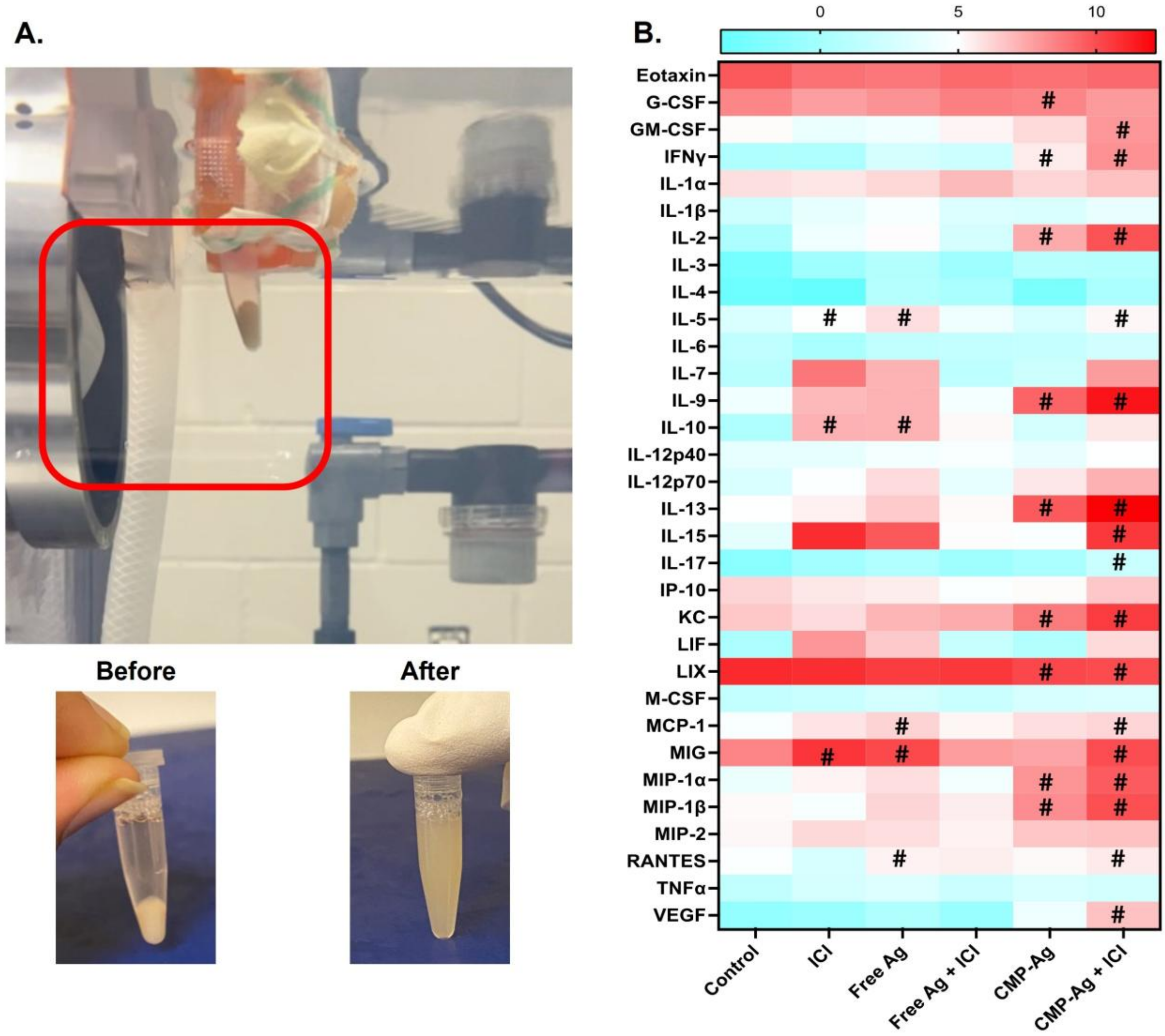

S4. CMPs efficiently adsorb and enrich tumor antigens, capturing diverse immune-relevant protein signatures. A) Tumor lysates from poorly immunogenic B16F10 (melanoma) and moderately immunogenic CT26 (colon carcinoma) cells generated by histotripsy (HT) were used to assess protein adsorption onto PCL, PLGA, and CMP microparticles. B) Heatmap of log-transformed cytokine/chemokine concentrations measured using a 32-plex Discovery assay.

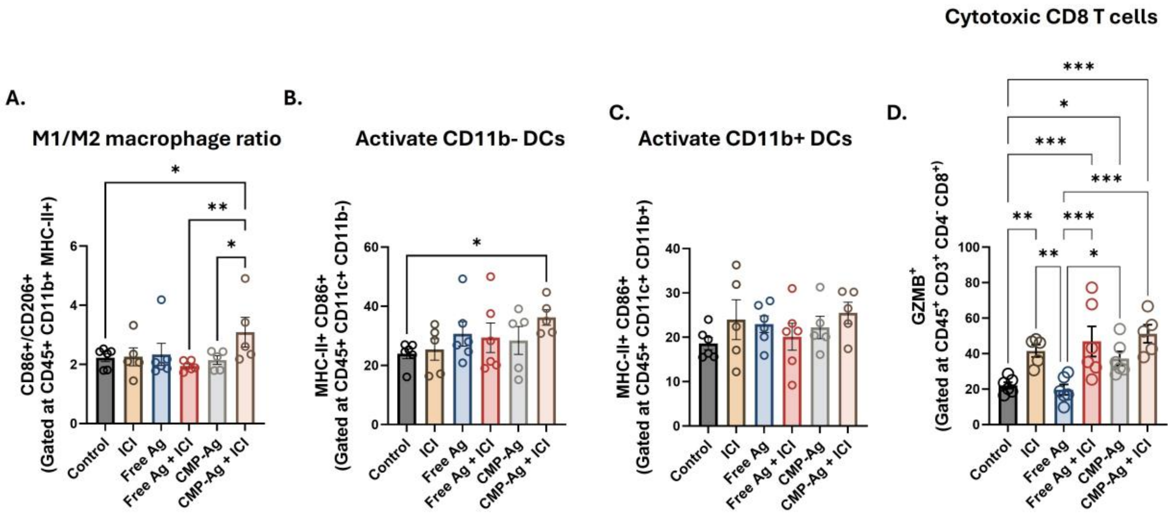

S5. B16F10 tumor-infiltrating DCs and CD8⁺ T cells following subcutaneous vaccination with CMPs adsorbed with HT-generated B16F10 antigens (CMP-Ag). A) Ratio of M1 macrophage (CD86+) to M2 macrophages (CD206+) numbers in treated tumors analyzed using flow cytometry. Cells were gated at CD45+ CD11c- CD11b+. B) Frequency of activated CD11b- DCs, MHC-II+ CD86+ double positive cells (% of parent population). C) Frequency of activated CD11b+ DCs, MHC-II+ CD86+ double positive cells (% of parent population). D) Frequency of GranzymeB (GZMB+) cytotoxic CD8+ T cells (gated at CD45+ CD3+ CD4-). Statistical test- One-way ANOVA with Tukey test. * $p<0.05$, ** $p<0.005$, *** $p<0.0005$, **** $p<0.0001$.

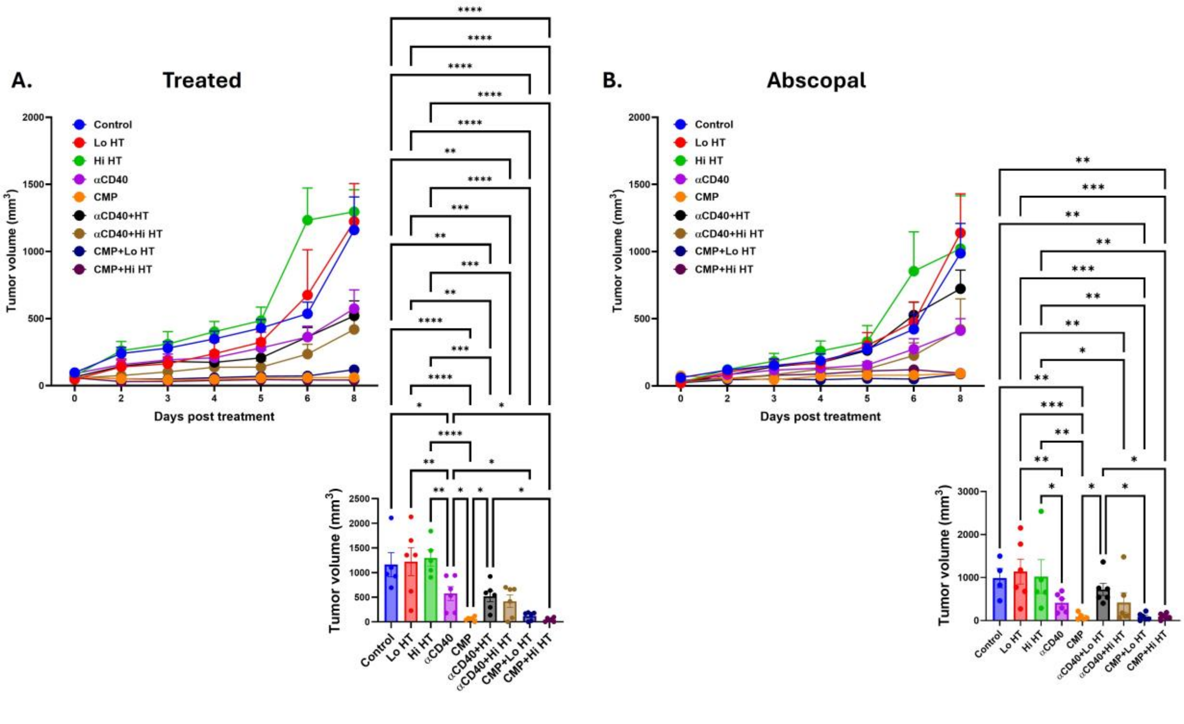

S6. Evaluation of local and abscopal effects of low HT (~10% tumor coverage) and high HT (>50% tumor coverage) in combination with CMP in poorly immunogenic B16F10 (melanoma) tumors (n = 6). A-B) B16F10 treated and abscopal (untreated) tumors following HT, αCD40, or CMP treatment, monitored up to 8 days post-treatment. Tumor volumes were

statistically analyzed on day 8. One-way ANOVA with Tukey Test used for D8 volumes analysis. *p < 0.05, **p < 0.005, ***p < 0.0005, ****p < 0.0001.

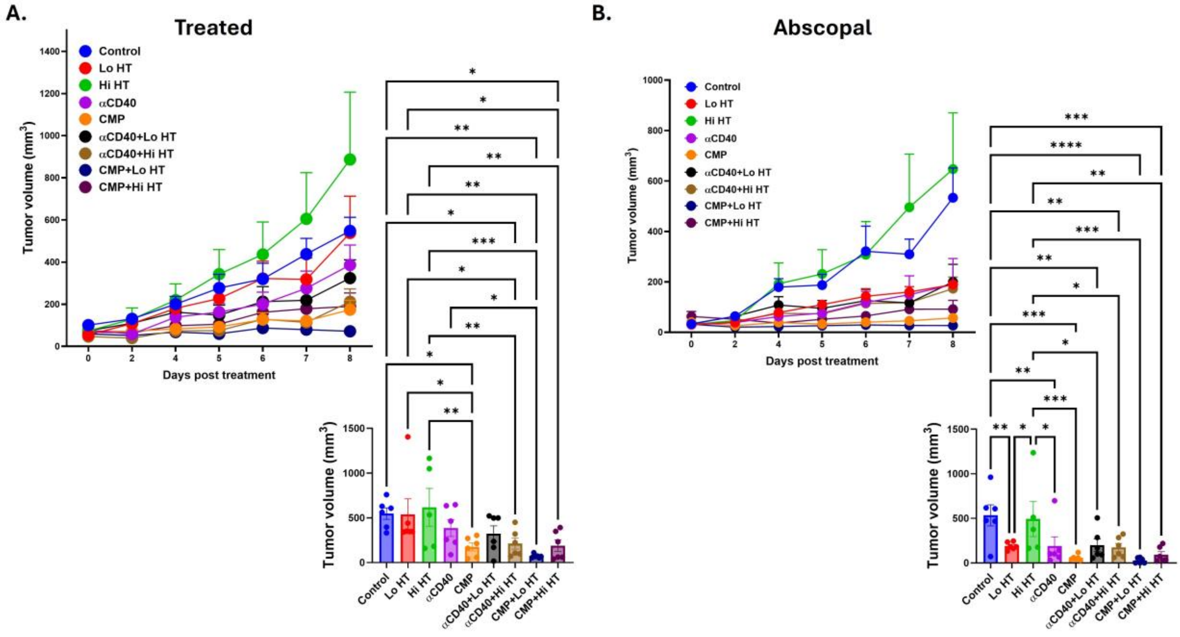

S7. Evaluation of local and abscopal effects of low HT (~10% tumor coverage) and high HT (>50% tumor coverage) in combination with CMP in moderately immunogenic CT26 (colon carcinoma) tumors (n = 6). A) CT26 treated and abscopal (untreated) tumors following HT, αCD40, or CMP treatment, monitored up to 8 days post-treatment. Tumor volumes were statistically analyzed on day 8. One-way ANOVA with Tukey Test used for D8 volumes analysis. *p < 0.05, **p < 0.005, ***p < 0.0005, ****p < 0.0001.

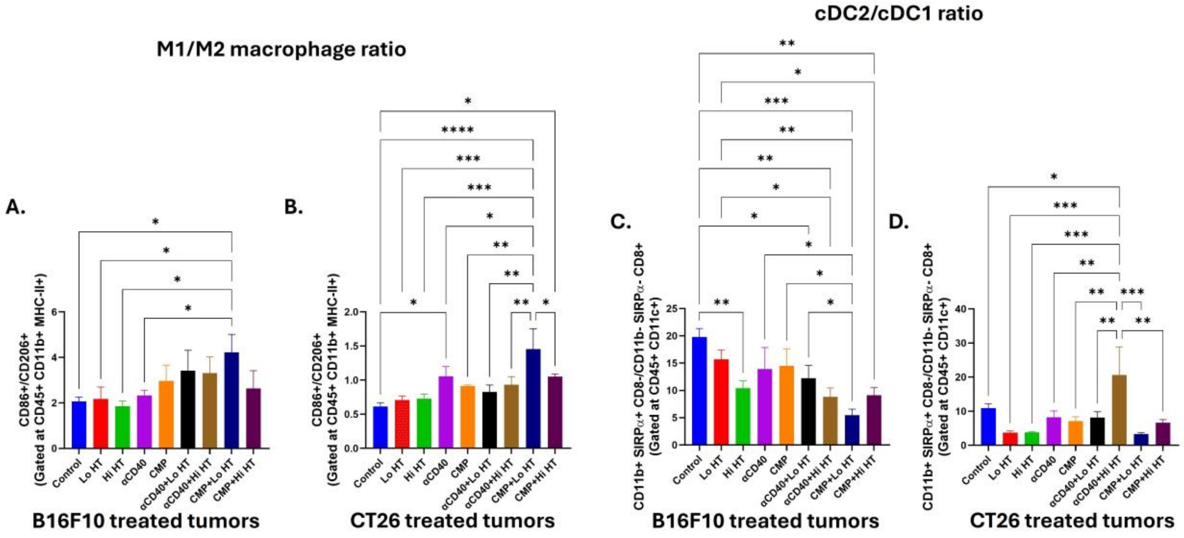

S8. Evaluation of macrophage and dendritic cell polarization in low (Lo) & Hi HT treated B16F10 & CT26 TME in combination with CMP treatment. A&B) M1 ($CD86^+$)/M2 ($CD206^+$) macrophage number ratio in treated B16F10 tumors (A) and CT26 tumors (B) analyzed using flow cytometry. Cells were gated at CD45+ CD11c- CD11b+. C&D) Ratio of cDC2 (CD11b+ SIRPα+ CD8-) to cDC1 (CD11b- SIRPα- CD8+) numbers in treated tumors,

gated at CD45+ CD11c+ cells in treated B16F10 tumors (C) and CT26 tumors (D). Statistical test for changes in immune cells were conducted using One-Way ANOVA with Fisher test, B16F10 tumor (n=5/6) and CT26 tumor (n=6) model data. * p<0.05, ** p<0.005, *** p<0.0005, **** p<0.0001.

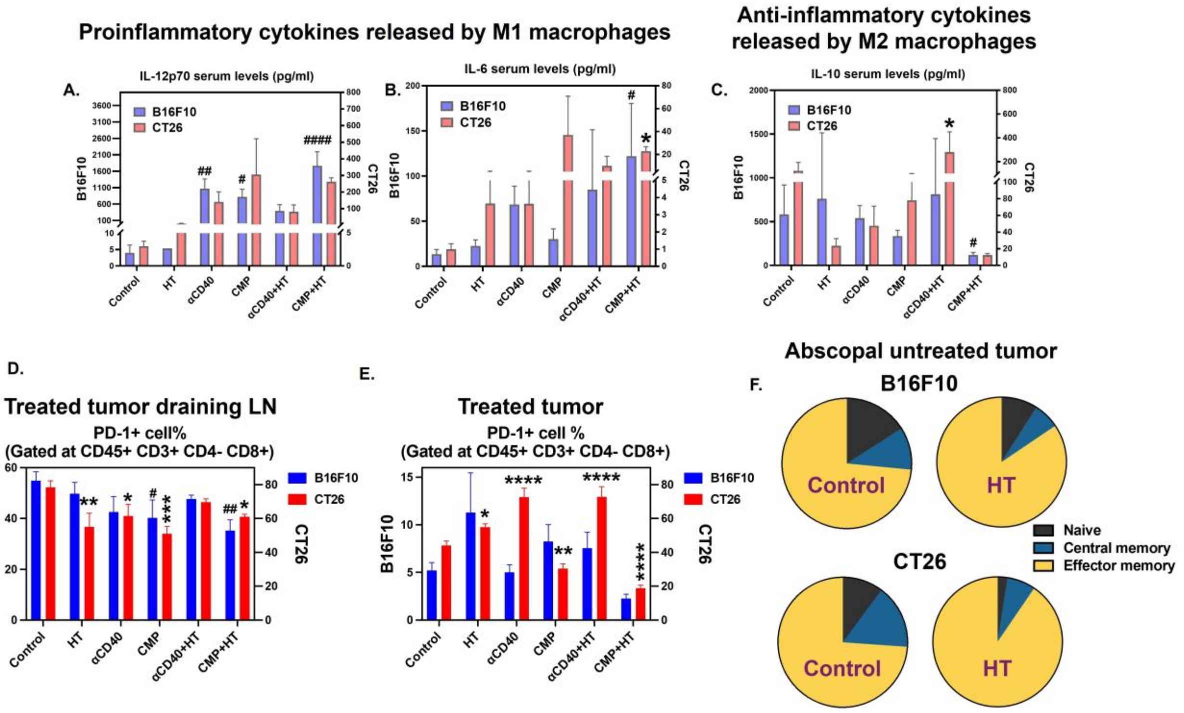

S9. CMP+HT treatment modulates M1/M2-associated serum cytokines and CD8⁺ T cell phenotypes in B16F10 and CT26 tumor-bearing mice. A-C) Serum levels of proinflammatory cytokines, IL-12p70 (A) & IL-6 (B), and anti-inflammatory cytokine, IL-10 (C), released by M1 macrophages and M2 macrophages respectively in B16F10 (n=6; blue) & CT26 (n=6; red) bearing mice 8 days post CMP+HT treatment. D&E) PD-1+ expression on CD8+ T cells (Gated at CD45+ CD3+ CD4-) accumulated in treated site TDLN (D) and treated tumors (E) of B16F10 (n=5/6; blue) and CT26 (n=6; red) models represented as % of CD8+ T cells. F) Changes in memory CD8 T cells infiltrating untreated abscopal tumors with HT treatment in B16F10 and CT26 tumor bearing mice. CD44- CD62L+ Naïve cells, CD44+ CD62L+ Central memory cells, CD44+ CD62L- Effector memory cells, all cells are gated at CD45+ CD3+ CD4- CD8+, part of whole graph used to show changes in percentage of different memory cell types averaged for treatment group. Statistical test for changes in immune cells for two tumor models were conducted separately using One-Way ANOVA with Fisher's LSD test. Significant changes for B16F10 model are shown as # and in CT26 model as *. */# p<0.05, **/## p<0.005, ***/### p<0.0005, ****/#### p<0.0001.

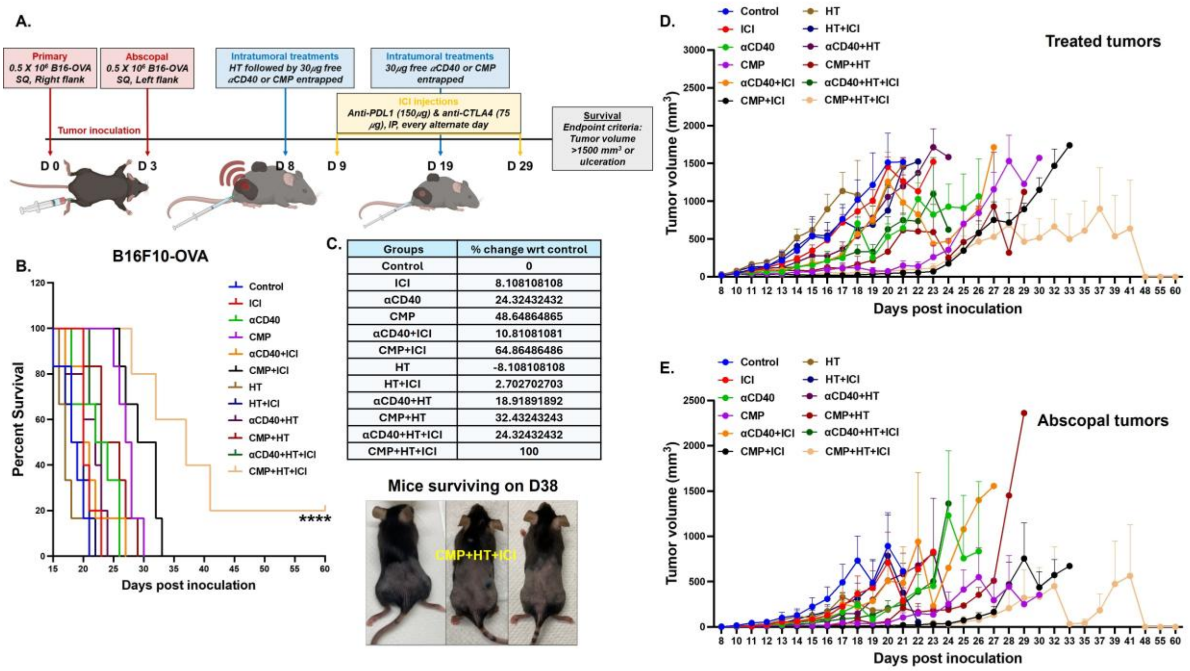

Groups	% change wrt control
Control	0
ICI	8.108108108
αCD40	24.32432432
CMP	48.64864865
αCD40+ICI	10.81081081
CMP+ICI	64.86486486
HT	-8.108108108
HT+ICI	2.702702703
αCD40+HT	18.91891892
CMP+HT	32.43243243
αCD40+HT+ICI	24.32432432
CMP+HT+ICI	100

S10. Survival of B16F10-OVA tumor–bearing mice treated with CMP+HT+ICI combination. A) Treatment timeline showing inoculation of bilateral subcutaneous B16F10-OVA tumors, followed by HT and CMP treatment combined with anti–PD-L1 and anti–CTLA-4 ICIs (n = 6) once tumors reached ~80 mm³. B) Survival in various treatment groups. C) Changes in median survival relative to the control group. Median survival for each treatment group was estimated by Kaplan-Meier statistical estimation, and percent change from control was calculated using ((Test – Control)/Control) × 100. Representative images of mice on day 38 post-inoculation, with survival observed only in the CMP+HT+ICI treatment group. D-E) Tumor growth curves of B16F10 tumors at the treated site (D) and abscopal site (E). Average tumor volume of each treatment group is shown until sacrifice (tumor volume >1500 mm³). Survival curves were compared using the log-rank (Mantel–Cox) test., **** $p<0.0001$.

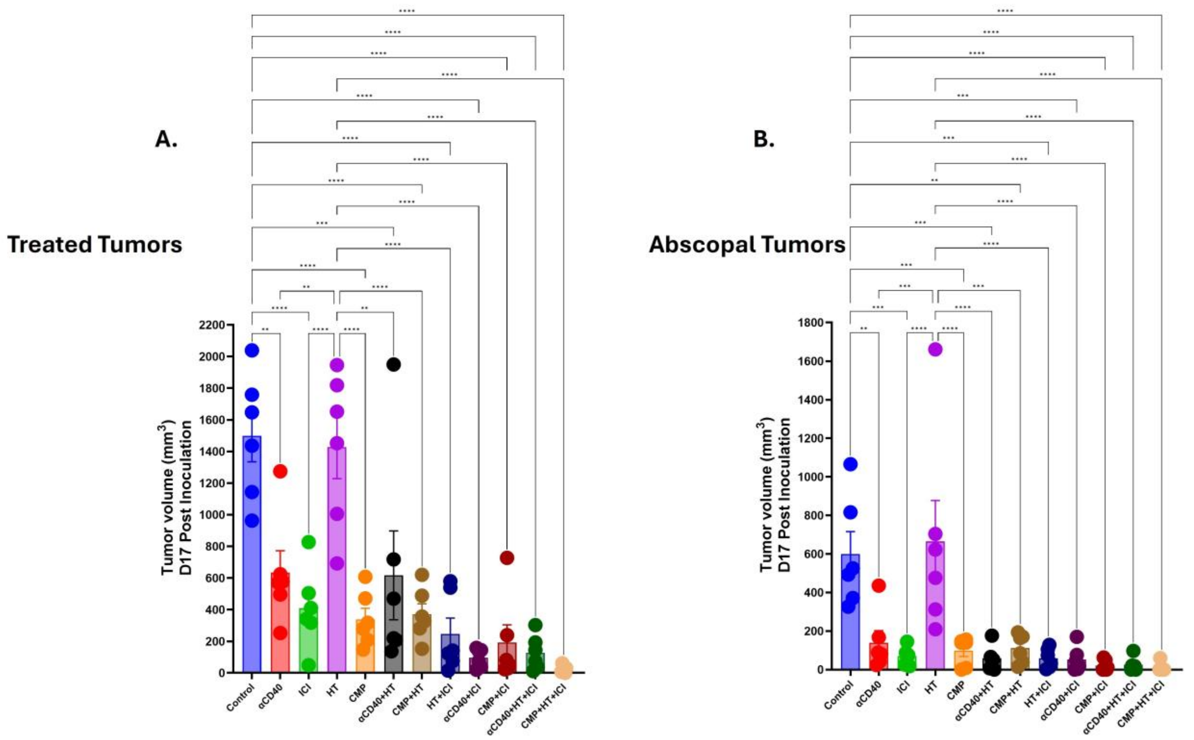

S11. Statistical analysis of tumor volume on day 17 after B16F10 primary (treated) tumor inoculation (supporting Fig. 8D and 8E). One-way ANOVA with Tukey post hoc test. * $p<0.05$, ** $p<0.005$, *** $p<0.0005$, **** $p<0.0001$.

Supplementary Tables:

Table S1. Frequencies of T cell subsets in tumor TDLNs, shown as % of parent population (mean ± SD) across treatment groups.

B16F10 model (n=5-6)		**CD8%**	**CD4%**	**PD1+ CD8 %**	**IL-2 CD8 %**
**CONTROL**	Average	14.70087058	63.24081949	54.99900732	6.943475556
	STDEV	6.285128241	7.054571112	6.750159593	4.753815037
**HT**	Average	12.40765455	63.91483787	49.83893392	12.56118055
	STDEV	4.923908706	7.675120987	9.763448918	14.09543589
**αCD40**	Average	16.59814472	51.8195358	42.60854751	6.63333553
	STDEV	4.392278414	4.749664135	13.5826586	2.6666633
**CMP**	Average	17.8993971	52.15192384	40.30150813	5.88590563
	STDEV	3.993315487	3.534602737	15.60538915	2.497106315
**αCD40 + HT**	Average	17.81711694	55.65127452	47.74179362	5.281017298
	STDEV	4.371380536	7.085271628	3.467460448	2.168703879
**CMP + HT**	Average	21.03205814	50.21483708	35.31915435	8.187464224
	STDEV	2.994018005	2.754166985	9.260691007	9.875972535
CT26 model (n=6)		**CD8%**	**CD4%**	**PD1+ CD8 %**	**IL-2 CD8 %**
**CONTROL**	Average	0.448925005	0.008877053	78.57025577	18.24811841
	STDEV	0.143818972	0.007041652	8.19461729	12.03410407
**HT**	Average	1.601221203	0.048453737	55.17647378	25.70897347
	STDEV	2.419287145	0.074479122	17.7350935	12.62693684
**αCD40**	Average	2.108876515	0.097665831	61.51729467	25.67227524
	STDEV	1.361908882	0.065801372	15.14657028	5.237788931
**CMP**	Average	5.058837407	0.273108812	51.14926692	12.41067895
	STDEV	2.080063127	0.116043855	9.296822893	5.120609889
**αCD40 + HT**	Average	2.748070401	0.174057341	69.76194797	26.0281851
	STDEV	2.66304985	0.164364298	4.254202359	6.752509408
**CMP + HT**	Average	6.578178284	0.438312513	61.08381378	31.96789138
	STDEV	7.356639141	0.376145324	3.144313892	13.70805074

Table S2: Frequencies of immune cells infiltrating untreated abscopal tumors, expressed as cells/mg tumor (mean ± SD) across treatment groups.

B16F10 model (n=5-6)		Myeloid cells			
		cDC2	cDC2 MHC-2+ CD86+	cDC1	cDC1 MHC-2+ CD86+
**CONTROL**	Average	26.1513832	12.48072924	3.18968393	0.107016054
	STDEV	18.23475816	9.379661165	4.620343485	0.187670286
**HT**	Average	18.17746496	8.58426573	1.14664303	0.024342036
	STDEV	11.70389179	6.827813199	0.775966655	0.033257311
**αCD40**	Average	76.82575195	40.5977781	3.205571802	0.134819356
	STDEV	41.09135113	15.35568764	1.417479656	0.051345718
**CMP**	Average	129.2022391	83.80336979	5.158013819	0.243449422
	STDEV	90.83685136	62.21598894	2.80783577	0.149902457
**αCD40 + HT**	Average	26.36737172	16.03544894	1.769744452	0.066002376
	STDEV	12.47414694	9.083837284	1.198031126	0.048782199
**CMP + HT**	Average	93.19709093	54.01827147	7.728378146	0.460287492
	STDEV	49.40077199	25.01005851	1.8559563	0.117551154

B16F10 model (n=5-6)		T cells			
		CD8 T cells	CD4 T cells	GZMB+ CD8 T cells	IL-2+ CD8 T cells
**CONTROL**	Average	0.564001359	98.56324255	0.076196644	0.130986826
	STDEV	0.484961679	128.0491946	0.040929002	0.08043224
**HT**	Average	0.313937143	33.0543152	0.030525602	0.086502138
	STDEV	0.276478449	27.41210372	0.022305396	0.050659663
**αCD40**	Average	1.499525968	161.8573508	0.175608125	0.398861748
	STDEV	0.570388208	110.9570894	0.138875323	0.281286562
**CMP**	Average	2.769281602	271.6743646	0.100916755	0.231598565
	STDEV	1.409181301	224.5253252	0.076545962	0.222180382
**αCD40 + HT**	Average	0.766811162	61.21001153	0.065268047	0.278834682
	STDEV	0.417096475	30.79405951	0.052239947	0.192235057
**CMP + HT**	Average	36.30872591	305.0741192	0.150990096	0.291747432
	STDEV	45.94871384	279.3653985	0.117863991	0.304906877

CT26 model (n=3-6)		Myeloid cells			
		cDC2	cDC2 MHC-2+ CD86+	cDC1	cDC1 MHC-2+ CD86+
**CONTROL**	Average	52.11780386	27.61390838	12.38994657	0.104523511
	STDEV	34.14639057	21.04979254	5.108514881	0.103280763
**HT**	Average	144.3977796	68.84518002	13.07954859	0.109661601
	STDEV	94.32662435	42.34469922	4.038572778	0.058603141
**αCD40**	Average	184.9063904	100.4986028	10.74221902	0.072927611

	STDEV	117.8012035	66.41343713	2.80764505	0.028748724
**CMP**	Average	393.0812723	217.0632533	15.42100046	0.129569307
	STDEV	165.0449954	113.5612148	2.901928596	0.027097765
**αCD40 + HT**	Average	316.3342567	179.4377029	16.44392138	0.141802109
	STDEV	264.5229378	146.7246002	11.00619227	0.073085168
**CMP + HT**	Average	408.5455468	199.3322863	15.41223016	0.131716985
	STDEV	76.00499862	43.66382098	2.046400045	0.020275598

CT26 model (n=3-6)		**T cells**			
		**CD8 T cells**	**CD4 T cells**	**GZMB+ CD8 T cells**	**IL-2+ CD8 T cells**
**CONTROL**	Average	7.895727318	131.0633332	4.042172112	3.376764844
	STDEV	4.942329823	137.6115656	2.114544499	1.9723814
**HT**	Average	12.55145994	193.5807751	4.054510467	3.627155528
	STDEV	7.838943776	126.9342035	1.099675295	0.661097405
**αCD40**	Average	33.27454848	319.0965408	11.8098024	11.52304252
	STDEV	27.11548652	306.0728338	5.722904951	7.52506021
**CMP**	Average	295.8941898	297.9530867	9.791824723	11.29414283
	STDEV	123.8115023	108.9390365	9.181255108	15.54036105
**αCD40 + HT**	Average	69.60113452	419.9661631	43.56406628	8.079533832
	STDEV	137.4269729	288.1935797	89.15350153	9.534991935
**CMP + HT**	Average	345.3155547	211.1108853	9.800049363	12.78169586
	STDEV	121.9628886	4.342293156	3.989542734	2.366421185

Table S3: Details of histotripsy parameter used for mice studies

**Parameter**	**Value**
Transducer	Single-element 1.5-MHz transducer (Alpinion VIFU2000)
Pulse duration	2ms
Pulse repetition frequency	5Hz
Duty cycle	1%
Power	600W (electrical input power)
Focal zone dimension (Lateral x Axial)	1x10mm
Treatment time per focal point	20s

Supplementary Movie:

S1. B-mode ultrasound imaging video showing hyperechogenicity during histotripsy treatment.
